# Supplementary material for: Associations between community cultural engagement and life satisfaction, mental distress and mental health functioning using data from the UK Household Longitudinal Study (UKHLS): are associations moderated by area deprivation?
Source: BMJ Open. 2021 Sep 3;11(9):e045512. doi: 10.1136/bmjopen-2020-045512 (PMC8420711; doi:10.1136/bmjopen-2020-045512)
Supplement: Supplementary data [file bmjopen-2020-045512supp001.pdf]

Appendix I

Cultural events

- A film at a cinema or other venue
- Exhibition or collection or art, photography or sculpture or a craft exhibition (not craft market)
- Event which included video or electronic art
- Event connected with books or writing
- Street arts or a public art display or installation (art in everyday surroundings, or an art work such as sculpture that is outdoors or in a public place)
- Carnival or culturally specific festival (for example, Mela, Baisakhi, Navrati, Feis)
- Circus (not animals)
- Play/drama, pantomime or a musical
- Opera/operetta
- Classical music performance
- Rock, pop or jazz performance
- Ballet
- Contemporary dance
- African people's dance or south Asian and Chinese dance

Museums/galleries and heritage sites visits

- Visit a museum or gallery
- Visit any of the following heritage sites:
    - A city or town with historic character
    - A historic building open to the public (non-religious)
    - A historic park or garden open to the public
    - A place connected with industrial history (e.g. an old factory, dockyard or mine) or historic transport system (e.g. an old ship or railway)
    - A historic place of worship attended as a visitor (not to worship)
    - A monument such as a castle, fort or ruin
    - A site of archaeological interest (e.g. Roman villa, ancient burial site)
    - A site connected with sports heritage (e.g. Wimbledon) (not visited for the purposes of watching sport)

Supplementary

Table S1: OLS regression estimating the association between community cultural engagement (CCE) at Wave 2 and life satisfaction at Wave 5 (weighted; N=14,783)

	M1: Basic model			M2: M1 + IMD & interaction terms			M3: M2 + Demographic factors			M4: M3 + Socio-economic position		
	Coef	95%CI	p-value	Coef	95%CI	p-value	Coef	95%CI	p-value	Coef	95%CI	p-value
Cultural events	**0.10**	**0.08 - 0.12**	**0.000**	**0.10**	**0.07 - 0.12**	**0.000**	**0.11**	**0.09 - 0.14**	**0.000**	**0.08**	**0.06 - 0.11**	**0.000**
20% most deprived area				**-0.32**	**-0.45 - -0.18**	**0.000**	**-0.26**	**-0.40 - -0.13**	**0.000**	**-0.16**	**-0.29 - -0.02**	**0.023**
20% least deprived area				**0.24**	**0.10 - 0.38**	**0.001**	**0.21**	**0.08 - 0.35**	**0.002**	**0.15**	**0.01 - 0.29**	**0.033**
(ref: medium levels of deprivation)												
Cultural events*20% most deprived area				-0.03	-0.08 - 0.03	0.353	-0.02	-0.08 - 0.03	0.443	-0.02	-0.07 - 0.04	0.485
Cultural events*20% least deprived area				-0.03	-0.08 - 0.02	0.264	-0.03	-0.08 - 0.02	0.203	-0.02	-0.07 - 0.03	0.352
Constant	**4.84**	**4.79 - 4.89**	**0.000**	**4.88**	**4.82 - 4.95**	**0.000**	**4.68**	**4.54 - 4.82**	**0.000**	**4.28**	**3.90 - 4.66**	**0.000**
R-squared	0.01			0.02			0.03			0.04		
Museums & heritage sites	**0.11**	**0.09 - 0.13**	**0.000**	**0.09**	**0.07 - 0.11**	**0.000**	**0.08**	**0.06 - 0.11**	**0.000**	**0.06**	**0.03 - 0.08**	**0.000**
20% most deprived area				**-0.34**	**-0.46 - -0.22**	**0.000**	**-0.30**	**-0.43 - -0.18**	**0.000**	**-0.19**	**-0.31 - -0.06**	**0.003**
20% least deprived area				**0.20**	**0.07 - 0.33**	**0.002**	**0.18**	**0.05 - 0.31**	**0.005**	0.11	-0.01 - 0.24	0.081
(ref: medium levels of deprivation)												
Museums & heritage sites*20% most deprived area				-0.01	-0.06 - 0.05	0.782	-0.01	-0.06 - 0.05	0.843	-0.00	-0.06 - 0.05	0.862
Museums & heritage sites*20% least deprived area				-0.02	-0.06 - 0.03	0.518	-0.02	-0.06 - 0.03	0.517	-0.01	-0.05 - 0.04	0.799
Constant	**4.85**	**4.80 - 4.90**	**0.000**	**4.91**	**4.85 - 4.97**	**0.000**	**4.85**	**4.72 - 4.98**	**0.000**	**4.36**	**3.98 - 4.74**	**0.000**
R-squared	0.01			0.02			0.03			0.04		

Note: M1 (basic model) included CCE. M2 additionally controlled for Index of Multiple Deprivation (IMD) and interaction terms (CCE*IMD). M3 additionally adjusted for demographic factors (age, gender, ethnicity, partnership status, presence of children under age 16, whether or not living alone, regional locations). M4 additionally adjusted for socio-economic position (educational levels, occupational socio-economic status, household monthly gross income, and housing tenure). Bold values denote statistical significance at the $p < 0.05$ level.

Table S2: OLS regression estimating the association between community cultural engagement (CCE) at Wave 2 and life satisfaction at Wave 5 (weighted; N=14,783)

	M1: Basic model			M2: M1 + IMD & interaction terms			M3: M2 + Demographic factors			M4: M3 + Socio-economic position		
	Coef	95%CI	p-value	Coef	95%CI	p-value	Coef	95%CI	p-value	Coef	95%CI	p-value
Cultural events	0.10	0.08 - 0.12	0.000	**0.10**	**0.08 - 0.12**	**0.000**	**0.11**	**0.09 - 0.13**	**0.000**	**0.08**	**0.06 - 0.10**	**0.000**
10% most deprived area				**-0.35**	**-0.53 - -0.17**	**0.000**	**-0.28**	**-0.46 - -0.10**	**0.002**	-0.16	-0.34 - 0.02	0.076
10% least deprived area				**0.28**	**0.09 - 0.47**	**0.004**	**0.26**	**0.07 - 0.45**	**0.008**	0.17	-0.02 - 0.37	0.074
(ref: medium levels of deprivation)												
Cultural events*10% most deprived area				-0.05	-0.14 - 0.03	0.217	-0.05	-0.13 - 0.03	0.251	-0.04	-0.12 - 0.04	0.348
Cultural events*10% least deprived area				-0.03	-0.09 - 0.04	0.418	-0.03	-0.10 - 0.03	0.281	-0.02	-0.09 - 0.04	0.471
Constant	4.84	4.79 - 4.89	0.000	**4.86**	**4.81 - 4.92**	**0.000**	**4.66**	**4.52 - 4.79**	**0.000**	**4.25**	**3.87 - 4.63**	**0.000**
R-squared	0.01			0.02			0.03			0.04		
Museums & heritage sites	0.11	0.09 - 0.13	0.000	**0.09**	**0.07 - 0.11**	**0.000**	**0.08**	**0.06 - 0.10**	**0.000**	**0.05**	**0.03 - 0.07**	**0.000**
10% most deprived area				**-0.41**	**-0.57 - -0.25**	**0.000**	**-0.38**	**-0.54 - -0.21**	**0.000**	**-0.24**	**-0.40 - -0.07**	**0.004**
10% least deprived area				**0.18**	**0.00 - 0.37**	**0.048**	0.16	-0.02 - 0.34	0.081	0.07	-0.11 - 0.25	0.462
(ref: medium levels of deprivation)												
Museums & heritage sites*10% most deprived area				-0.00	-0.08 - 0.07	0.921	-0.00	-0.08 - 0.08	0.981	0.00	-0.07 - 0.08	0.902
Museums & heritage sites*10% least deprived area				0.01	-0.05 - 0.07	0.789	0.01	-0.05 - 0.07	0.807	0.02	-0.04 - 0.08	0.505
Constant	4.85	4.80 - 4.90	0.000	**4.90**	**4.84 - 4.95**	**0.000**	**4.83**	**4.71 - 4.96**	**0.000**	**4.34**	**3.96 - 4.72**	**0.000**
R-squared	0.01			0.02			0.03			0.04		

Note: M1 (basic model) included CCE. M2 additionally controlled for Index of Multiple Deprivation (IMD) and interaction terms (CCE*IMD). M3 additionally adjusted for demographic factors (age, gender, ethnicity, partnership status, presence of children under age 16, whether or not living alone, regional locations). M4 additionally adjusted for socio-economic position (educational levels, occupational socio-economic status, household monthly gross income, and housing tenure). Bold values denote statistical significance at the p < 0.05 level.

Table S3: OLS regression estimating the association between community cultural engagement (CCE) at Wave 2 and mental distress (GHQ-12) at Wave 5 (weighted; N=14,783)

	M1: Basic model			M2: M1 + IMD & interaction terms			M3: M2 + Demographic factors			M4: M3 + Socio-economic position		
	Coef	95%CI	p-value	Coef	95%CI	p-value	Coef	95%CI	p-value	Coef	95%CI	p-value
Cultural events	**-0.02**	**-0.03 - -0.02**	**0.000**	**-0.02**	**-0.02 - -0.01**	**0.000**	**-0.02**	**-0.03 - -0.02**	**0.000**	**-0.02**	**-0.02 - -0.01**	**0.000**
20% most deprived area				**0.09**	**0.05 - 0.13**	**0.000**	**0.08**	**0.04 - 0.12**	**0.000**	**0.05**	**0.01 - 0.09**	**0.012**
20% least deprived area				**-0.07**	**-0.11 - -0.03**	**0.000**	**-0.07**	**-0.11 - -0.03**	**0.001**	**-0.05**	**-0.09 - -0.01**	**0.007**
(ref: medium levels of deprivation)												
Cultural events*20% most deprived area				-0.01	-0.03 - 0.00	0.070	-0.01	-0.03 - 0.00	0.105	-0.01	-0.03 - 0.00	0.118
Cultural events*20% least deprived area				0.01	-0.01 - 0.02	0.383	0.01	-0.01 - 0.02	0.376	0.00	-0.01 - 0.02	0.613
Constant	**1.97**	**1.96 - 1.99**	**0.000**	**1.96**	**1.94 - 1.98**	**0.000**	**2.01**	**1.96 - 2.05**	**0.000**	**2.14**	**2.03 - 2.25**	**0.000**
R-squared	0.01			0.01			0.03			0.04		
Museums & heritage sites	**-0.02**	**-0.03 - -0.02**	**0.000**	**-0.02**	**-0.02 - -0.01**	**0.000**	**-0.01**	**-0.02 - -0.01**	**0.000**	**-0.01**	**-0.02 - -0.00**	**0.013**
20% most deprived area				**0.08**	**0.04 - 0.12**	**0.000**	**0.08**	**0.04 - 0.12**	**0.000**	**0.05**	**0.01 - 0.08**	**0.014**
20% least deprived area				**-0.06**	**-0.10 - -0.02**	**0.002**	**-0.06**	**-0.10 - -0.02**	**0.002**	**-0.04**	**-0.08 - -0.01**	**0.025**
(ref: medium levels of deprivation)												
Museums & heritage sites*20% most deprived area				-0.01	-0.03 - 0.00	0.108	-0.01	-0.03 - 0.00	0.096	-0.01	-0.03 - 0.00	0.151
Museums & heritage sites*20% least deprived area				0.00	-0.01 - 0.01	0.924	0.00	-0.01 - 0.01	0.862	-0.00	-0.02 - 0.01	0.758
Constant	**1.97**	**1.95 - 1.98**	**0.000**	**1.95**	**1.93 - 1.97**	**0.000**	**1.97**	**1.93 - 2.00**	**0.000**	**2.12**	**2.01 - 2.23**	**0.000**
R-squared	0.01			0.01			0.03			0.04		

Note: M1 (basic model) included CCE. M2 additionally controlled for Index of Multiple Deprivation (IMD) and interaction terms (CCE*IMD). M3 additionally adjusted for demographic factors (age, gender, ethnicity, partnership status, presence of children under age 16, whether or not living alone, regional locations). M4 additionally adjusted for socio-economic position (educational levels, occupational socio-economic status, household monthly gross income, and housing tenure). Bold values denote statistical significance at the $p < 0.05$ level.

Table S4: OLS regression estimating the association between community cultural engagement (CCE) at Wave 2 and mental distress (GHQ-12) at Wave 5 (weighted; N=14,783)

	M1: Basic model			M2: M1 + IMD & interaction terms			M3: M2 + Demographic factors			M4: M3 + Socio-economic position		
	Coef	95%CI	p-value	Coef	95%CI	p-value	Coef	95%CI	p-value	Coef	95%CI	p-value
Cultural events	**-0.02**	**-0.03 - -0.02**	**0.000**	**-0.02**	**-0.03 - -0.01**	**0.000**	**-0.03**	**-0.03 - -0.02**	**0.000**	**-0.02**	**-0.03 - -0.01**	**0.000**
10% most deprived area				**0.09**	**0.04 - 0.15**	**0.001**	**0.08**	**0.02 - 0.13**	**0.005**	0.04	-0.01 - 0.10	0.118
10% least deprived area				**-0.10**	**-0.15 - -0.04**	**0.001**	**-0.09**	**-0.15 - -0.04**	**0.001**	**-0.07**	**-0.12 - -0.01**	**0.013**
(ref: medium levels of deprivation)												
Cultural events*10% most deprived area				0.00	-0.02 - 0.02	0.883	0.00	-0.02 - 0.03	0.702	0.00	-0.02 - 0.03	0.714
Cultural events*10% least deprived area				0.01	-0.01 - 0.03	0.155	0.01	-0.00 - 0.03	0.150	0.01	-0.01 - 0.03	0.326
Constant	**1.97**	**1.96 - 1.99**	**0.000**	**1.97**	**1.95 - 1.98**	**0.000**	**2.02**	**1.98 - 2.06**	**0.000**	**2.15**	**2.04 - 2.26**	**0.000**
R-squared	0.01			0.01			0.03			0.04		
Museums & heritage sites	**-0.02**	**-0.03 - -0.02**	**0.000**	**-0.02**	**-0.02 - -0.01**	**0.000**	**-0.02**	**-0.02 - -0.01**	**0.000**	**-0.01**	**-0.02 - -0.00**	**0.001**
10% most deprived area				**0.12**	**0.07 - 0.17**	**0.000**	**0.11**	**0.06 - 0.16**	**0.000**	**0.07**	**0.02 - 0.12**	**0.004**
10% least deprived area				**-0.08**	**-0.13 - -0.02**	**0.006**	**-0.08**	**-0.13 - -0.02**	**0.004**	**-0.05**	**-0.11 - -0.00**	**0.044**
(ref: medium levels of deprivation)												
Museums & heritage sites*10% most deprived area				-0.02	-0.04 - 0.01	0.137	-0.02	-0.04 - 0.00	0.113	-0.02	-0.04 - 0.01	0.140
Museums & heritage sites*10% least deprived area				0.01	-0.01 - 0.02	0.521	0.01	-0.01 - 0.03	0.430	0.00	-0.01 - 0.02	0.734
Constant	**1.97**	**1.95 - 1.98**	**0.000**	**1.95**	**1.94 - 1.97**	**0.000**	**1.97**	**1.93 - 2.01**	**0.000**	**2.12**	**2.01 - 2.23**	**0.000**
R-squared	0.01			0.01			0.02			0.04		

Note: M1 (basic model) included CCE. M2 additionally controlled for Index of Multiple Deprivation (IMD) and interaction terms (CCE*IMD). M3 additionally adjusted for demographic factors (age, gender, ethnicity, partnership status, presence of children under age 16, whether or not living alone, regional locations). M4 additionally adjusted for socio-economic position (educational levels, occupational socio-economic status, household monthly gross income, and housing tenure). Bold values denote statistical significance at the $p < 0.05$ level.

Table S5: OLS regression estimating the association between community cultural engagement (CCE) at Wave 2 and mental health functioning (SF-12) at Wave 5 (weighted; N=14,783)

	M1: Basic model			M2: M1 + IMD & interaction terms			M3: M2 + Demographic factors			M4: M3 + Socio-economic position		
	Coef	95%CI	p-value	Coef	95%CI	p-value	Coef	95%CI	p-value	Coef	95%CI	p-value
Cultural events	**0.10**	**0.10 - 0.11**	**0.000**	**0.10**	**0.09 - 0.11**	**0.000**	**0.08**	**0.07 - 0.09**	**0.000**	**0.05**	**0.04 - 0.06**	**0.000**
20% most deprived area				**-0.18**	**-0.24 - -0.12**	**0.000**	**-0.19**	**-0.25 - -0.13**	**0.000**	**-0.10**	**-0.16 - -0.04**	**0.001**
20% least deprived area				**0.17**	**0.11 - 0.24**	**0.000**	**0.16**	**0.10 - 0.22**	**0.000**	**0.10**	**0.04 - 0.16**	**0.001**
(ref: medium levels of deprivation)												
Cultural events*20% most deprived area				0.01	-0.01 - 0.03	0.380	0.01	-0.01 - 0.03	0.463	0.01	-0.01 - 0.03	0.505
Cultural events*20% least deprived area				**-0.02**	**-0.04 - -0.00**	**0.033**	-0.01	-0.03 - 0.01	0.217	-0.00	-0.02 - 0.02	0.890
Constant	**3.53**	**3.50 - 3.55**	**0.000**	**3.55**	**3.51 - 3.58**	**0.000**	**4.01**	**3.95 - 4.06**	**0.000**	**3.81**	**3.66 - 3.97**	**0.000**
R-squared	0.05			0.07			0.12			0.16		
Museums & heritage sites	**0.08**	**0.07 - 0.09**	**0.000**	**0.07**	**0.06 - 0.09**	**0.000**	**0.07**	**0.06 - 0.08**	**0.000**	**0.05**	**0.04 - 0.06**	**0.000**
20% most deprived area				**-0.16**	**-0.21 - -0.10**	**0.000**	**-0.17**	**-0.22 - -0.11**	**0.000**	**-0.07**	**-0.13 - -0.02**	**0.008**
20% least deprived area				**0.16**	**0.10 - 0.22**	**0.000**	**0.16**	**0.10 - 0.21**	**0.000**	**0.10**	**0.05 - 0.16**	**0.000**
(ref: medium levels of deprivation)												
Museums & heritage sites*20% most deprived area				-0.00	-0.02 - 0.02	0.989	-0.00	-0.03 - 0.02	0.760	-0.01	-0.03 - 0.01	0.419
Museums & heritage sites*20% least deprived area				-0.02	-0.04 - 0.00	0.072	-0.01	-0.03 - 0.01	0.190	-0.00	-0.02 - 0.02	0.805
Constant	**3.60**	**3.57 - 3.62**	**0.000**	**3.62**	**3.59 - 3.64**	**0.000**	**4.10**	**4.04 - 4.15**	**0.000**	**3.84**	**3.68 - 4.00**	**0.000**
R-squared	0.03			0.05			0.11			0.16		

Note: M1 (basic model) included CCE. M2 additionally controlled for Index of Multiple Deprivation (IMD) and interaction terms (CCE*IMD). M3 additionally adjusted for demographic factors (age, gender, ethnicity, partnership status, presence of children under age 16, whether or not living alone, regional locations). M4 additionally adjusted for socio-economic position (educational levels, occupational socio-economic status, household monthly gross income, and housing tenure). Bold values denote statistical significance at the $p < 0.05$ level.

Table S6: OLS regression estimating the association between community cultural engagement (CCE) at Wave 2 and mental health functioning (SF-12) at Wave 5 (weighted; N=14,783)

	M1: Basic model			M2: M1 + IMD & interaction terms			M3: M2 + Demographic factors			M4: M3 + Socio-economic position		
	Coef	95%CI	p-value	Coef	95%CI	p-value	Coef	95%CI	p-value	Coef	95%CI	p-value
Cultural events	**0.10**	**0.10 - 0.11**	**0.000**	**0.10**	**0.09 - 0.11**	**0.000**	**0.08**	**0.07 - 0.09**	**0.000**	**0.05**	**0.04 - 0.06**	**0.000**
10% most deprived area				**-0.21**	**-0.29 - -0.13**	**0.000**	**-0.21**	**-0.29 - -0.13**	**0.000**	**-0.11**	**-0.19 - -0.03**	**0.006**
10% least deprived area				**0.24**	**0.16 - 0.32**	**0.000**	**0.22**	**0.14 - 0.29**	**0.000**	**0.14**	**0.06 - 0.21**	**0.000**
(ref: medium levels of deprivation)												
Cultural events*10% most deprived area				-0.01	-0.04 - 0.03	0.693	-0.01	-0.04 - 0.02	0.500	-0.01	-0.04 - 0.02	0.597
Cultural events*10% least deprived area				**-0.04**	**-0.06 - -0.01**	**0.004**	**-0.03**	**-0.05 - -0.00**	**0.043**	-0.01	-0.03 - 0.02	0.484
Constant	**3.53**	**3.50 - 3.55**	**0.000**	**3.54**	**3.51 - 3.57**	**0.000**	**3.99**	**3.93 - 4.04**	**0.000**	**3.80**	**3.64 - 3.96**	**0.000**
R-squared	0.05			0.07			0.11			0.16		
Museums & heritage sites	**0.08**	**0.07 - 0.09**	**0.000**	**0.07**	**0.07 - 0.08**	**0.000**	**0.07**	**0.06 - 0.08**	**0.000**	**0.05**	**0.04 - 0.06**	**0.000**
10% most deprived area				**-0.23**	**-0.30 - -0.16**	**0.000**	**-0.24**	**-0.31 - -0.17**	**0.000**	**-0.12**	**-0.19 - -0.05**	**0.001**
10% least deprived area				**0.21**	**0.13 - 0.28**	**0.000**	**0.20**	**0.13 - 0.27**	**0.000**	**0.12**	**0.06 - 0.18**	**0.000**
(ref: medium levels of deprivation)												
Museums & heritage sites*10% most deprived area				0.00	-0.03 - 0.04	0.899	0.00	-0.03 - 0.04	0.818	0.00	-0.03 - 0.03	0.958
Museums & heritage sites*10% least deprived area				**-0.03**	**-0.05 - -0.00**	**0.034**	-0.02	-0.04 - 0.00	0.111	-0.00	-0.03 - 0.02	0.769
Constant	**3.60**	**3.57 - 3.62**	**0.000**	**3.61**	**3.59 - 3.64**	**0.000**	**4.09**	**4.04 - 4.14**	**0.000**	**3.83**	**3.68 - 3.99**	**0.000**
R-squared	0.03			0.05			0.11			0.16		

Note: M1 (basic model) included CCE. M2 additionally controlled for Index of Multiple Deprivation (IMD) and interaction terms (CCE*IMD). M3 additionally adjusted for demographic factors (age, gender, ethnicity, partnership status, presence of children under age 16, whether or not living alone, regional locations). M4 additionally adjusted for socio-economic position (educational levels, occupational socio-economic status, household monthly gross income, and housing tenure). Bold values denote statistical significance at the p < 0.05 level.
