# Supplementary material for: Associations between community cultural engagement and life satisfaction, mental distress and mental health functioning using data from the UK Household Longitudinal Study (UKHLS): are associations moderated by area deprivation?
Source: BMJ Open. 2021 Sep 3;11(9):e045512. doi: 10.1136/bmjopen-2020-045512 (PMC8420711; doi:10.1136/bmjopen-2020-045512)
Supplement: Author's manuscript [file bmjopen-2020-045512.draft_revisions.pdf]

BMJ Open is committed to open peer review. As part of this commitment we make the peer review history of every article we publish publicly available.

When an article is published we post the peer reviewers' comments and the authors' responses online. We also post the versions of the paper that were used during peer review. These are the versions that the peer review comments apply to.

The versions of the paper that follow are the versions that were submitted during the peer review process. They are not the versions of record or the final published versions. They should not be cited or distributed as the published version of this manuscript.

BMJ Open is an open access journal and the full, final, typeset and author-corrected version of record of the manuscript is available on our site with no access controls, subscription charges or pay-per-view fees (http://bmjopen.bmj.com).

If you have any questions on BMJ Open's open peer review process please email info.bmjopen@bmj.com

BMJ Open

Longitudinal associations between community cultural engagement and wellbeing: are effects moderated by area deprivation?

Journal:	*BMJ Open*
Manuscript ID	bmjopen-2020-045512
Article Type:	Original research
Date Submitted by the Author:	05-Oct-2020
Complete List of Authors:	Mak, Hei Wan; University College London, Department of Behavioural Science and Health<br>Coulter, Rory; University College London, Department of Geography<br>Fancourt, Daisy; University College London Research Department of Epidemiology and Public Health, Department of Behavioural Science and Health
Keywords:	MENTAL HEALTH, PUBLIC HEALTH, SOCIAL MEDICINE

SCHOLARONE™
Manuscripts

I, the Submitting Author has the right to grant and does grant on behalf of all authors of the Work (as defined in the below author licence), an exclusive licence and/or a non-exclusive licence for contributions from authors who are: i) UK Crown employees; ii) where BMJ has agreed a CC-BY licence shall apply, and/or iii) in accordance with the terms applicable for US Federal Government officers or employees acting as part of their official duties; on a worldwide, perpetual, irrevocable, royalty-free basis to BMJ Publishing Group Ltd ("BMJ") its licensees and where the relevant Journal is co-owned by BMJ to the co-owners of the Journal, to publish the Work in this journal and any other BMJ products and to exploit all rights, as set out in our licence.

The Submitting Author accepts and understands that any supply made under these terms is made by BMJ to the Submitting Author unless you are acting as an employee on behalf of your employer or a postgraduate student of an affiliated institution which is paying any applicable article publishing charge ("APC") for Open Access articles. Where the Submitting Author wishes to make the Work available on an Open Access basis (and intends to pay the relevant APC), the terms of reuse of such Open Access shall be governed by a Creative Commons licence – details of these licences and which Creative Commons licence will apply to this Work are set out in our licence referred to above.

Other than as permitted in any relevant BMJ Author's Self Archiving Policies, I confirm this Work has not been accepted for publication elsewhere, is not being considered for publication elsewhere and does not duplicate material already published. I confirm all authors consent to publication of this Work and authorise the granting of this licence.

For peer review only

Longitudinal associations between community cultural engagement and wellbeing: are effects moderated by area deprivation?

Hei Wan Mak[1], Rory Coulter[2], Daisy Fancourt[1]

[1]Department of Behavioural Science and Health, University College London, London, UK

[2]Department of Geography, University College London, London, UK

Corresponding author: Daisy Fancourt (d.fancourt@ucl.ac.uk)

Keywords: community engagement; area deprivation; mental wellbeing; life satisfaction

Word count: 3392

For peer review only

Abstract

Objectives. To examine whether the association between engagement in community cultural assets (attendance at cultural events, and visiting museums and heritage sites) and subsequent wellbeing (life satisfaction, mental distress and mental health functioning) is moderated by neighbourhood deprivation.

Design. Data were drawn from Understanding Society: The UK Household Longitudinal Study (UKHLS) Waves 2 and 5. Participating households' addresses were geocoded into statistical neighbourhood zones categorised according to their level of area deprivation.

Setting. General population.

Participants. UK general adult population, with a total sample of 17,948.

Main outcome measures. Life satisfaction was measured with a 7-point scale (1-completely unsatisfied to 7-completely satisfied). Mental distress was measured using the GHQ-12 (General Health Questionnaire). Mental health functioning was measured using SF-12 (12-Item Short Form Health Survey).

Results. Using OLS regression, we found that engagement in cultural assets was consistently and positively associated with subsequent life satisfaction (attending cultural events: coef=0.10, 95%CI=0.06, 0.13, p<0.001; visiting museums & heritage sites: coef=0.06, 95%CI=0.03, 0.10, p<0.001) and mental health functioning (attending cultural events: coef=0.11, 95%CI=0.09, 0.13, p<0.001; visiting museums & heritage sites: coef=0.08, 95%CI=0.06, 0.10, p<0.001) and negatively associated with mental distress (attending cultural events: coef=-0.07, 95%CI=-0.09, -0.04, p<0.001; visiting museums & heritage sites: coef=-0.04, 95%CI=-0.06, -0.02, p<0.001). Importantly, such associations were independent of individuals' demographic background, socio-economic characteristics and regional location. Our paper also shows that relationship between cultural events and wellbeing was stronger in deprived areas.

Conclusions. This study shows that engagement in community cultural assets are associated with better wellbeing, with some evidence of moderation that individuals in areas of high deprivation potentially may benefit more from the engagement. This provides support for the validity of place-based funding schemes that involve investment in areas of higher deprivation to improve engagement rates, which in turn help promote better wellbeing amongst individuals in such areas.

Strengths and limitations of this study

For peer review only

- This is first large, population-based study to investigate whether the longitudinal association between community cultural engagement and mental wellbeing varies with local deprivation.
- This study was based on a nationally representative sample with a 3-year follow up period and the analysis controlled for a wide range of important confounding factors.
- However, as this was an observational study, causality cannot be established.
- The Index of Multiple Deprivation is comprised of various components (e.g. living environment, income and employment deprivation) as a proxy of neighbourhood effect.

Ethics approval

The University of Essex Ethics Committee has approved all data collection on Understanding Society: The UK Household Longitudinal Study (UKHLS) main study and innovation panel waves, including asking consent for all data linkages except to health records. Respondents aged 16 or above provided written consent to participate.

Availability of data and materials

UKHLS data is available from the UK Data Service https://discover.ukdataservice.ac.uk/catalogue/?sn=6614. Data documentation is available from the Understanding Society website https://www.understandingsociety.ac.uk/documentation. Deprivation data are captured through linking lower-layer super output areas (LSOA) data from the UKHLS with data from the Index of Multiple Deprivation (IMD) provided by the Office for National Statistics Postcodes Directory (ONSPD).

Acknowledgement

DF is supported by the Wellcome Trust [205407/Z/16/Z]. HWM is funded through the AHRC project HEARTS [AH/P005888/1]. This project is also supported by UCL Grand Challenges Award “Environment and Wellbeing” [551987/100/156425 33002] and ESRC WELLCOMM project [ES/T006994/1].

Author Contributions

HWM conducted the data management, data analyses and provided input on the manuscript. RC and DF assisted with analytical issues, provided input on the analytical scheme and the manuscript. All authors are responsible for reported research and have participated in the

For peer review only

concept and design, analysis and interpretation of data, and drafting and revising of the manuscript.

For peer review only

Introduction

The role of community cultural engagement (CCE) in health and wellbeing has received increased attention across a number of disciplines over the past two decades. CCE can include going to cultural events (such as the theatre, opera, concerts or exhibitions) and visiting museums and heritage sites (including visiting a city or town with historic character), and has been associated with improvements in wellbeing [1–3], slower declines in cognition [4], reduced levels of isolation and loneliness [5](Todd et al, 2017), enhanced social wellbeing in the community [6] and lower mortality rates [7,8]. These benefits have been found amongst

healthy individuals as well as people with mental health problems, dementia, substance use addiction, carers, families living in deprived areas, asylum seekers and isolated adults [9–11].

Unfortunately, previous studies have suggested a social and geographical gradient in cultural engagement, in which the engagement rate is higher amongst people living in more affluent areas [12–14]. Certainly, CCE relies on cultural assets (i.e. tangible spaces, buildings or organisations) being available within communities, and most of the community cultural assets are unevenly distributed across the UK in ways which co-vary with deprivation [15–17]. In addition, even when cultural assets are available, the characteristics of neighbourhoods — such as the living environment, local facilities and services, social connections and networks, levels of accessibility, and the safety of the area — have been identified as specific factors that can affect the engagement rate [12,14,18,19]. As such some people may have greater, safer, and easier opportunities to engage with culture (the 'contextual effect'). But it is worth noting that this effect may be less obvious in those deprived parts of England that lie in close proximity to more affluent areas with greater availability of assets [15–17].

Alternatively, it has also been shown that, independent of area deprivation, people living in particular areas tend to cluster together according to their demographics (e.g. age, ethnicity), socio-economic position (ability to afford housing) and lifestyle or cultural preferences. This can lead to the acquisition of common norms, values, and economic and cultural capital, which may lead to different behavioural patterns of CCE within specific neighbourhoods (known as the 'compositional effect') [20,21]. Compositional effects have been demonstrated for CCE in previous studies, where neighbourhoods can be defined by their characteristics such as "cosmopolitan" areas or "student" areas have been identified as having particularly strong patterns of CCE relative, say, to industrious communities areas [13].

However, variations in people's CCE behaviours may not be simply a matter of contextual and compositional effects. It is plausible that the place people live is not only a *predictor* of CCE, but also a *moderator* of the association between CCE and health outcomes. There is evidence of similar social and geographical gradients in CCE and health, with many studies showing spatially variable associations between area deprivation and poor health outcomes, although results have been complex and not entirely consistent [22–24]. Indeed, many studies when examining health inequality have incorporated geographical data and have suggested that

For peer review only

unequal access to health-promoting environments, such as the availability of and accessibility to cultural and artistic activities, could contribute to the spatial health divide [12,25]. This is particularly relevant to areas of deprivation where educational level, employment rate and living standard are lower and mental health problems are more common[26]. Therefore, it is plausible that bringing cultural resources to deprived areas and improving community infrastructure could have a greater impact on people's wellbeing in these areas than in affluent areas, as in deprived areas there may otherwise be limited assets and opportunities to build positive mental health.

In light of this, it is relevant to explore whether place is important not just in predicting levels of CCE but also in moderating the relationship between CCE and health outcomes. Understanding whether there is any moderation is crucial and relevant to current public health strategies and interventions such as 'social prescribing' schemes and place-based funding streams for the cultural sector. These are predicated on the belief that increasing the local availability of assets and their usage could lead to increased CCE and improved health outcomes [27–32]. But it remains unclear whether investment in cultural assets in different locations holds equal potential for positively influencing health.

Therefore, in this paper, we used a large longitudinal and nationally-representative sample of adults to examine whether the association between engagement in community cultural assets and subsequent wellbeing (operationalised as life satisfaction, mental distress and mental health functioning) is moderated by geographical deprivation in the origin location. In particular, given that differential exposure to risks affects health differently, we explored whether individuals living in areas of high deprivation who are at higher risks of experiencing poorer mental health gain the same or even greater wellbeing benefits from CCE as individuals living in areas of low deprivation.

Data & Methods

We used data from Understanding Society: The UK Household Longitudinal Study (UKHLS), which is a continuation of the long-running British Household Panel Survey (BHPS). UKHLS follows over 50,000 individuals from 30,000 households annually [33] and collects rich information on engagement in cultural events and museums and heritage (a list of cultural events is presented in Appendix 1). Crucially, the survey also collects a suite of measures of

For peer review only

self-reported individual wellbeing. The University of Essex Ethics Committee approved the study and participants provided informed consent.

In order to investigate the role of area deprivation, we used geo-coded Understanding Society data in which participating households' addresses have been positioned into a number of spatial zoning systems (e.g. administrative and census statistical geographies). For our analysis, we extracted a sample of adults living in England who responded to the Wave 2 (2010/12; response rate=84%), where data on engagement were first available, and Wave 5 (2013/15; response rate=85%) interviews (unweighted N=23,315). Only respondents who answered across all measures were considered (unweighted N=22,519).

Patient and public involvement

This study used publicly available secondary data from the UK Data Service (https://www.ukdataservice.ac.uk/). Patients and the public were not involved.

Measures

We defined neighbourhoods as 2011 census Lower Layer Super Output Areas (LSOA). LSOAs are designed for the consistent reporting of small area statistics in England and Wales (ONS- Census geography). Using the LSOA geocodes, we attached the 2015 Index of Multiple Deprivation (IMD 2015), which measures the relative deprivation of small areas across seven domains: income, employment, health deprivation and disability, education, skills and training, crime, barriers to housing and service and living environment. Our main analysis is based on the IMD decile rank values which we used as a continuous measure for our main analyses. In addition, we tested using an ordered categorical measure of deprivation to capture non-linear effects and stratification between the most and least deprived areas. To do this we coded IMD into (1) the 20% most deprived areas, (2) 20% least deprived and (3) the remaining intermediate neighbourhoods.

For CCE, we focused on attendance at cultural events; including going to the theatre, concerts, opera and exhibition (a full list of cultural events is provided in Appendix), and visiting museums and heritage sites. CCE was measured in Wave 2. Frequency of engagement with these activities was categorised as "not once in the last 12 months", "once in the last 12 months", "twice in the last 12 months", "less often than once a month but at least 3 or 4 times

For peer review only

a year", "less often than once a week but at least once a month" and "at least once a week". The variables are treated as continuous measures.

We explored three outcome wellbeing measures in Wave 5: life satisfaction, mental distress and mental health functioning. Life satisfaction was measured through asking respondents how satisfied they felt with their life overall, with responses ranging from 1 (completely unsatisfied) to 7 (completely satisfied). Mental distress was measured using the GHQ-12 (General Health Questionnaire); a screening device identifying psychiatric disorders in the general population. The GHQ-12 self-reported questionnaire includes 12 4-point items (such as sleeping problems, overall happiness and depressive symptoms). The scale was standardised with a mean of 0 and a standard deviation of 1, with higher scores indicating a greater incidence of mental distress. Mental health functioning was measured using SF-12 (12-Item Short Form Health Survey); a well-validated survey that was designed to measure respondents' general quality of life. The 12-item survey contains eight indicators: physical functioning, role limitations due to physical health problems, bodily pain, general health, vitality, social functioning, role limitations due to emotional problems and mental health [34]. The scale was standardised with higher scores indicating better levels of mental health functioning.

In our analysis, we controlled for broad regional variations (North vs Midlands vs South) as well as demographic and socio-economic variables in Wave 2 that could predict wellbeing. Demographic control variables included age, gender, ethnicity (White vs Asian/Asian British vs Black/Black British vs mixed/others), partnership status (married or in cohabitation vs single and never married or never in civil partnership vs separated or divorced or widowed), presence of child(ren) under the age of 16, and whether respondents were living alone. Socio-economic controls included educational level (university degree vs Advanced (A-levels) exams/higher education (e.g. a Higher Education Certificate/BTEC) vs passed General Certificate of Secondary Education-GCSE vs unrecognised qualification/no qualification), occupational socio-economic status (managerial or professional vs intermediate or small employment or own account vs lower supervision or technician or semi-routine or routine vs not in employment (e.g. unemployed or retired or students)), household income, and tenure (house owner vs social rent vs private rent).

For peer review only

Statistical analysis

17.5% of the data were missing, therefore multiple imputation by chained equations was employed to create 50 imputed datasets, using the exposure and confounding variables in the models as predictors. Results were nearly identical when using just the available data and so in what follows we present the imputed analyses for greater statistical power. The analyses were carried out using Stata 16.

To understand whether the longitudinal relationship between CCE (x) and mental wellbeing (y) varied with Wave 2 area deprivation (the potential moderator), we used sequentially constructed OLS regression models. We also tested using different measures of area deprivation (i.e. the original ten-fold measure and a three-fold measure using 20% categories) to check the robustness of our results. All models were weighted using inverse probability weights derived from the Wave 2 longitudinal weights supplied with UKHLS. These weights have been tailored to the analysis sample and should correct our estimates to take into account differential sample selection and retention probabilities [35]. As further robustness checks, all analyses were replicated with another three-fold measure using 10% IMD threshold (i.e. 10% most deprived areas vs medium level of deprivation vs 10% least deprived areas) and on a cross-sectional dataset in which mental wellbeing was measured in Wave 2.

Results

(Table 1)

Table 1 reports weighted descriptive statistics of the original and imputed samples. There are no significant differences in terms of the frequency of CCE. In our imputed sample, the average age was 47 years, 53% were female, and over 90% were of white ethnicity. In relation to socio-economic position, 35% of the respondents had a university degree, 27% were in managerial/professional roles and 70% owned a house.

(Tables 2-4)

Life satisfaction

After adjusting for individual demographic factors and socio-economic positions, both types of CCE were associated with a greater level of life satisfaction (attending cultural events: coef=0.10, 95%CI=0.06, 0.13, p<0.001; visiting museums & heritage sites: coef=0.06,

For peer review only

95%CI=0.03, 0.10, p<0.001) (Table 2). There was no evidence that this association was moderated by area deprivation when using the continuous measure. However, when using the three-fold 20% measure, we found an interaction effect of cultural events*20% most deprived areas, which shows that the rate of increase in life satisfaction that accompanies CCE is stronger amongst people living in deprived areas (Fig. 1). These results were replicated when using the three-fold 10% measure (Supplementary Table S1 & Fig. S1).

Mental distress (GHQ-12)

When accounting for individual demographic and socio-economic characteristics, attending cultural events was associated with lower levels of mental distress (coef=-0.07, 95%CI=-0.09, -0.04, p<0.001) and so was visiting museums and heritage sites (coef=-0.04, 95%CI=-0.06, -0.02, p<0.001) (Table 3). There was no evidence of moderation by area deprivation across any of the two approaches or with the three-fold 10% measure (Supplementary Table S1).

Mental health functioning (SF-12)

After adjusting for demographic background and socio-economic position, both types of CCE were associated with a higher level of subsequent mental health functioning (attending cultural events: coef=0.11, 95%CI=0.09, 0.13, p<0.001; visiting museums & heritage sites: coef=0.08, 95%CI=0.06, 0.10, p<0.001) (Table 4). There was some indication of moderation of the association between cultural events and mental health functioning by area deprivation, both when using the continuous measure or three-fold 20% measure (Fig. 2 shows the moderation with the three-fold 20% measure). No moderation effect was found with the three-fold 10% measure (Supplementary Table S1).

Sensitivity analyses

When predicting life satisfaction, mental distress and mental health functioning at Wave 2 rather than Wave 5, the longitudinal results were largely replicated. After adjusting for individual demographic and socio-economic factors, higher levels of CCE were associated with greater life satisfaction (attending cultural events: coef=0.11, 95%CI=0.08, 0.13, p<0.001; visiting museums & heritage sites: coef=0.08, 95%CI=0.06, 0.11, p<0.001). We also found a moderation effect of cultural attendance*20% least deprived areas (coef=-0.04, 95%CI=-0.07, -0.01, p<0.05), but not with museums & heritage sites (Supplementary Table S2). Our analysis also shows that CCE was negatively related to mental health distress (attending cultural

For peer review only

events: coef=-0.07, 95%CI=-0.09, -0.06, p<0.001; visiting museums & heritage sites: coef=-0.05, 95%CI=-0.06, -0.03, p<0.001). No moderation effect was found for mental health distress (Supplementary Table S3). Finally, higher levels of CCE were associated with better mental health functioning (attending cultural events: coef=0.11, 95%CI=0.10, 0.13, p<0.001; visiting museums & heritage sites: coef=0.08, 95%CI=0.07, 0.10, p<0.001). There was an indication of interaction effect of cultural attendance*20% most deprived areas (coef=0.02, 95%CI=0.00, 0.04, p<0.05), but not with museums & heritage sites (Supplementary Table S4).

Discussion

This is the first large population-based study to investigate whether the longitudinal association between CCE (attending cultural events or visiting museums and heritages sites) and mental wellbeing (including life satisfaction, mental distress and mental health functioning) varies with local deprivation. In line with previous research, our results show that engagement in cultural assets was consistently and positively associated with subsequent life satisfaction and mental health functioning and negatively associated with mental distress [3]. Importantly, this paper found that such associations were independent of individuals' demographic background, socio-economic characteristics and regional locations. This suggests that, while social and geographical factors could influence the engagement rate [12–14,18,36], CCE is consistently associated with higher wellbeing regardless of where people live (even amongst people living in deprived areas).

Notably, our analysis shows some indications of interactions between cultural events and area deprivation. In particular, we found that the rates of growth in life satisfaction and mental health functioning that accompany CCE are stronger amongst people living in deprived areas. This suggests that individuals who live in highly deprived areas may benefit the most from attending cultural events in terms of positive mental health. This provides an important insight by suggesting that when individuals living in deprived areas are offered the opportunities to engaging in CCE, they could potentially experience greater improvements in life satisfaction and mental wellbeing than those living in wealthy areas who have already been able to benefit from higher levels of engagement (possibly since childhood) and who usually enjoy other social benefits (e.g. higher social positions) that help maintain their mental wellbeing. Despite this, deprivation appears to be a consistent barrier to engagement [13,14,18]. People living in

For peer review only

deprived areas are less likely to engage in CCE not simply because of their personal background and characteristics (e.g. lower educational attainment and occupational position), but because of the areas they reside in offer less cultural opportunities (e.g. unsafe, culturally deprived, undesirable) [14,18]. Consequently, unequal access to the arts may inhibit people living in deprived communities from enjoying the benefits provided by CCE and hence exacerbate social, cultural and health inequalities.

The results of this study have clear implications for the design and roll-out of place-based programmes for CCE that operate under the assumption that investing in areas of high deprivation and low cultural opportunity could improve wellbeing levels. One example of such a scheme is the UK 'Creative People and Places' funded by Arts Council England. The data presented here suggest that such schemes have the potential to achieve their aims as there is some indication that the benefits of CCE may be more pronounced in more deprived areas. But there are mixed implications for other schemes that aim to signpost individuals to existing community cultural activities to improve health and wellbeing, as occurs in 'social prescribing'. While such an approach clearly has some potential to achieve its health aims in the same way as place-based funding schemes, social prescribing schemes rely on the availability of community cultural assets within communities. It has been suggested that CCE can be increased in areas with more cultural opportunity structures and in areas with high accessibility to cultural infrastructures, especially for groups that have often been excluded from CCE [12]. The success of such schemes will therefore rely on their ability to overcome the psychological, logistical or structural barriers that could limit individuals' engagement within more deprived areas.

A key strength of this paper is that it is based on a nationally representative sample, with a 3-year follow up period. Our analyses controlled for a wide range of potentially confounding factors and were consistent across three different measures of area deprivation. However, our study is not without its limitations. First, this is an observational study and causality cannot be established. But a number of previous studies have already confirmed that CCE can be causally linked to wellbeing through randomised interventions [1,2], and the generalisability of such findings has been suggested through causal inference analyses of longitudinal data in other papers [3]. It is, however, unclear why there was not a consistent association between area deprivation and mental wellbeing in our analyses, which goes against some previous

For peer review only

research findings (e.g. Bellis et al., 2012[37]). It is possible that after considering all individual demographic backgrounds and socio-economic characteristics might have weakened the effect of area deprivation, given that personal characteristics, cultural consumption, lifestyle and inbuilt environment are interrelated. As another limitation, the Index of Multiple Deprivation is comprised of various components (e.g. living environment, income and employment deprivation) as a proxy of neighbourhood effect. But it will be important to understand from future studies how other neighbourhood characteristics (e.g. demographic structure and population density) may also influence the engagement level. Relatedly, future study may be needed to replicate the research on young people's data, given that the moderating effects of area deprivation may be more noticeable during childhood when young people are less able to travel to engage in cultural activities outside of their immediate area of dwelling. Finally, due to data limitations, we were unable to identify whether CCE was recent or more long-standing. It is likely that people who engage in culture and heritage over long periods of time may experience a greater level of mental wellbeing.

Conclusion

Our study provides insights into the role of place in long-term relationships between engagement in community cultural assets (both cultural events and museums and heritage sites) and mental wellbeing. Specifically, a higher CCE rate is associated with greater levels of life satisfaction and mental health functioning and with reduced mental distress. Notably, such relationships are independent of individual demographic and socio-economic characteristics, and CCE is consistently associated with higher wellbeing regardless of where people live. As such, individuals living in areas of high deprivation can experience the same wellbeing benefits from CCE as individuals living in areas of low deprivation. Further, there is also some evidence of moderation, with individuals in areas of high deprivation potentially even able to benefit more from CCE in terms of life satisfaction and mental health functioning. However, this does not mitigate the problem that individuals in areas of high deprivation are less likely to engage in CCE. This, therefore, suggests the validity of place-based funding schemes that involve investment in areas of higher deprivation to improve engagement rates and suggests that such schemes could help to promote higher levels of wellbeing amongst individuals in such areas. Further intervention studies testing this finding further experimentally are encouraged.

For peer review only

Table 1: Descriptive statistics of the original and imputed sample [weighted]

	Original sample (N=14,815)	Imputed sample (N=17,948)
	Proportion/ Mean (SE)	Proportion/ Mean (SE)
Community cultural engagement		
Cultural events[1]	2.22 (0.01)	2.17 (0.01)
Museums & heritage sites[1]	1.96 (0.01)	1.90 (0.01)
Mental health and wellbeing, Wave 5		
Life satisfaction[2]	5.06 (0.01)	5.03 (0.01)
Mental distress (GHQ-12; standardised)	-0.01 (0.01)	-0.00 (0.01)
Mental health functioning (SF-12; standardised)	0.01 (0.01)	-0.01 (0.01)
Demographic backgrounds		
Age (mean)	47.0	46.8 (0.16)
Gender		
Female	52.9	52.8
Male	47.2	47.2
Ethnicity		
White	91.2	92.0
Asian/Asian British	5.02	4.51
Black/Black British	1.94	1.74
Mixed/other	1.80	1.71
Living alone		
No	85.0	84.9
Yes	15.0	15.1
Partnership status		
Single and never married	21.9	22.8
Married or in cohabitation	64.3	63.3
Separated or divorced or widowed	13.9	13.9
Responsible for child(ren) under 16		
No	83.7	83.4
Yes	16.3	16.6
Regions		

For peer review only

North (North East, North West and Yorkshire and the Humber)	28.1	28.4
Midlands (East Midlands and West Midlands)	18.7	19.1
South (London, South East, South West and East)	53.2	52.5
Socio-economic position (SEP)		
Educational levels		
University degree	35.1	34.5
Advanced (higher degree/A-level)	20.3	20.5
GCSE or equivalent	21.3	20.9
Unrecognised/no qualification	23.2	24.1
Socio-economic status (SES)		
Managerial/professional	27.5	26.5
Intermediate/small employment/own account	15.8	15.4
lower supervision or lower technician/semi-routine and routine	23.0	23.8
Not working (incl. retired, full-time student)	33.8	34.3
Logged personal income monthly	7.39 (0.01)	7.38 (0.01)
Housing tenure		
House owner	70.5	69.7
Social rent	15.9	17.0
Private rent	13.6	13.3
Levels of area deprivation[3]	4.58 (0.03)	4.82 (0.02)

Notes: [1]A six-point scale, ranging from "not once in the last 12 months", "once in the last 12 months", "twice in the last 12 months", "less often than once a month but at least 3 or 4 times a year", "less often than once a week but at least once a month" to "at least once a week". [2]Life satisfaction was measured using a scale from 1 "completely dissatisfied" to 7 "completely satisfied". [3]Levels of area deprivation was derived from the Index of Multiple Deprivation, which has a scale from 1 "most deprived 10%" to 10 "least deprived 10%".

For peer review only

Table 2: OLS regression estimating the association between community cultural engagement (CCE) and life satisfaction at Wave 5 (weighted; N=17,948)

	Cultural events				Museums & heritage sites			
	Basic model	+ IMD, interaction terms	+ Demographic factors	+ Socio-economic positions	Basic model	+ IMD, interaction terms	+ Demographic factors	+ Socio-economic positions
IMD as a continuous variable								
CCE	0.10 (0.08, 0.12)***	0.12 (0.08, 0.16)***	0.14 (0.11, 0.18)***	0.10 (0.06, 0.13)***	0.11 (0.09, 0.12)***	0.12 (0.09, 0.15)***	0.11 (0.07, 0.14)***	0.06 (0.03, 0.10)***
IMD		0.01 (-0.01, 0.02)	0.01 (-0.01, 0.02)	0.01 (-0.01, 0.02)		0.00 (-0.01, 0.02)	0.00 (-0.01, 0.02)	-0.00 (-0.02, 0.01)
CCE*IMD		-0.00 (-0.01, 0.00)	-0.00 (-0.01, 0.00)	-0.00 (-0.01, 0.00)		-0.00 (-0.01, 0.00)	-0.00 (-0.01, 0.00)	-0.00 (-0.01, 0.00)
Constant	4.81 (4.76, 4.86)***	4.78 (4.69, 4.88)***	4.55 (4.40, 4.71)***	4.11 (3.75, 4.48)***	4.83 (4.78, 4.87)***	4.82 (4.74, 4.91)***	4.77 (4.63, 4.91)***	4.23 (3.87, 4.60)***
F-statistics	121.43	40.91	23.40	22.82	151.26	51.19	19.81	21.68
Prob > F	0.000	0.000	0.000	0.000	0.000	0.000	0.000	0.000
Using 20% thresholds								
CCE	0.10 (0.08, 0.12)***	0.09 (0.07, 0.12)***	0.11 (0.08, 0.13)***	0.06 (0.04 0.09)***	0.11 (0.09, 0.12)***	0.10 (0.08, 0.12)***	0.09 (0.06, 0.11)***	0.05 (0.02, 0.07)***
IMD (ref: medium)								
20% most deprived		-0.07 (-0.18, 0.04)	-0.09 (-0.20, 0.02)	-0.09 (-0.20, 0.01)		-0.03 (-0.13, 0.06)	-0.04 (-0.14, 0.06)	-0.03 (-0.13, 0.07)
20% least deprived		-0.03 (-0.17, 0.11)	-0.03 (-0.18, 0.11)	-0.05 (-0.19, 0.10)		-0.04 (-0.17, 0.09)	-0.05 (-0.18, 0.08)	-0.05 (-0.18, 0.08)
CCE*IMD								
CCE*20% most deprived		0.03 (-0.01, 0.07)	0.04 (-0.00, 0.08)	0.04 (0.00, 0.08)*		0.02 (-0.02, 0.06)	0.02 (-0.02, 0.06)	0.02 (-0.02, 0.06)
CCE*20% least deprived		0.00 (-0.05, 0.06)	0.00 (-0.05, 0.06)	0.01 (-0.05, 0.06)		0.01 (-0.05, 0.06)	0.01 (-0.04, 0.06)	0.01 (-0.04, 0.06)
Constant	4.81 (4.76, 4.86)***	4.84 (4.77, 4.90)***	4.62 (4.49, 4.76)***	4.18 (3.82, 4.54)***	4.83 (4.78, 4.87)***	4.84 (4.78, 4.90)***	4.79 (4.66, 4.91)***	4.25 (3.89, 4.60)***

For peer review only

F-statistics	121.43	24.88	20.83	21.32	151.26	30.96	17.47	20.07
Prob > F	0.000	0.000	0.000	0.000	0.000	0.000	0.000	0.000

Table 3: OLS regression estimating the association between community cultural engagement (CCE) and mental health distress (GHQ-12) at Wave 5 (weighted; N=17,948)

	Cultural events				Museums & heritage sites			
	Basic model	+ IMD, interaction terms	+ Demographic factors	+ Socio-economic positions	Basic model	+ IMD, interaction terms	+ Demographic factors	+ Socio-economic positions
IMD as a continuous variable								
CCE	-0.06 (-0.07, -0.05)***	-0.08 (-0.10, -0.05)***	-0.09 (-0.11, -0.06)***	-0.07 (-0.09, -0.04)***	-0.05 (-0.06, -0.04)***	-0.06 (-0.08, -0.04)***	-0.06 (-0.08, -0.04)***	-0.04 (-0.06, -0.02)***
IMD		-0.01 (-0.02, 0.00)	-0.01 (-0.02, 0.00)	-0.01 (-0.02, 0.00)		-0.00 (-0.01, 0.01)	-0.00 (-0.01, 0.01)	-0.00 (-0.01, 0.01)
CCE*IMD		0.00 (-0.00, 0.01)	0.00 (-0.00, 0.01)	0.00 (-0.00, 0.01)		0.00 (-0.00, 0.01)	0.00 (-0.00, 0.01)	0.00 (-0.00, 0.01)
Constant	0.13 (0.10, 0.16)***	0.16 (0.10, 0.23)***	0.19 (0.09, 0.29)***	0.42 (0.18, 0.65)**	0.10 (0.07, 0.13)***	0.11 (0.05, 0.16)***	0.05 (-0.04, 0.14)	0.33 (0.09, 0.56)**
F-statistics	100.92	35.49	21.91		89.02	30.30	18.56	16.38
Prob > F	0.000	0.000	0.000	0.000	0.000	0.000	0.000	0.000
Using 20% thresholds								
CCE	-0.06 (-0.07, -0.05)***	-0.06 (-0.07, -0.04)***	-0.07 (-0.08, -0.05)***	-0.04 (-0.06, -0.03)***	-0.05 (-0.06, -0.04)***	-0.05 (-0.06, -0.03)***	-0.04 (-0.06, -0.03)***	-0.03 (-0.04, -0.01)***

For peer review only

**IMD (ref: medium)**								
**20% most deprived**		0.02 (-0.05, 0.09)	0.03 (-0.05, 0.10)	0.03 (-0.04, 0.10)		0.02 (-0.05, 0.08)	0.02 (-0.05, 0.08)	0.01 (-0.05, 0.08)
**20% least deprived**		-0.04 (-0.13, 0.05)	-0.04 (-0.13, 0.05)	-0.04 (-0.13, 0.06)		0.01 (-0.08, 0.09)	0.00 (-0.08, 0.09)	0.01 (-0.08, 0.09)
**CCE*IMD**								
**CCE*20% most deprived**		-0.01 (-0.04, 0.02)	-0.01 (-0.04, 0.01)	-0.02 (-0.04, 0.01)		-0.01 (-0.04, 0.01)	-0.01 (-0.04, 0.01)	-0.01 (-0.04, 0.01)
**CCE*20% least deprived**		0.01 (-0.02, 0.05)	0.01 (-0.02, 0.05)	0.01 (-0.02, 0.05)		-0.01 (-0.04, 0.03)	-0.01 (-0.04, 0.03)	-0.01 (-0.04, 0.02)
**Constant**	0.13 (0.10, 0.16)***	0.13 (0.09, 0.17)***	0.15 (0.07, 0.24)***	0.38 (0.15, 0.61)**	0.10 (0.07, 0.13)***	0.10 (0.06, 0.14)***	0.04 (-0.04, 0.12)	0.32 (0.09, 0.55)**
**F-statistics**	100.92	20.75	19.20	15.91	89.02	18.13	16.53	15.24
**Prob > F**	0.000	0.000	0.000	0.000	0.000	0.000	0.000	0.000

Table 4: OLS regression estimating the association between community cultural engagement (CCE) and mental health functioning (SF-12) at Wave 5 (weighted; N=17,948)

	Cultural events				Museums & heritage sites			
	Basic model	+ IMD, interaction	+ Demographic	+ Socio-economic	Basic model	+ IMD, interaction	+ Demographic	+ Socio-economic

For peer review only

		terms	factors	positions		terms	factors	positions
IMD as a continuous variable								
CCE	0.16 (0.15, 0.18)***	0.19 (0.17, 0.21)***	0.17 (0.14, 0.19)***	0.11 (0.09, 0.13)***	0.13 (0.12, 0.14)***	0.14 (0.12, 0.16)***	0.13 (0.11, 0.15)***	0.08 (0.06, 0.10)***
IMD		0.01 (0.00, 0.03)*	0.01 (0.00, 0.03)*	0.01 (0.00, 0.02)*		0.00 (-0.01, 0.02)	0.00 (-0.01, 0.01)	0.00 (-0.01, 0.01)
CCE*IMD		-0.01 (-0.01, -0.00)*	-0.01 (-0.01, -0.00)**	-0.01 (-0.01, -0.00)***		-0.00 (-0.01, 0.00)	-0.00 (-0.01, 0.00)	-0.00 (-0.01, 0.00)
Constant	-0.37 (-0.41, -0.34)***	-0.44 (-0.51, -0.38)***	0.26 (0.16, 0.35)***	0.01 (-0.23, 0.24)	-0.26 (-0.29, -0.23)***	-0.29 (-0.35, -0.23)***	0.48 (0.39, 0.57)***	0.13 (-0.10, 0.35)
F-statistics	724.63	246.56	97.43	92.82	482.61	161.69	90.21	92.27
Prob > F	0.000	0.000	0.000	0.000	0.000	0.000	0.000	0.000
Using 20% thresholds								
CCE	0.16 (0.15, 0.18)***	0.16 (0.15, 0.18)***	0.14 (0.12, 0.15)***	0.08 (0.06, 0.09)***	0.13 (0.12, 0.14)***	0.13 (0.11, 0.14)***	0.11 (0.10, 0.13)***	0.07 (0.05, 0.08)***
IMD (ref: medium)								
20% most deprived		-0.07 (-0.15, 0.01)	-0.07 (-0.14, 0.01)	-0.07 (-0.15, -0.00)*		-0.04 (-0.11, 0.03)	-0.05 (-0.12, 0.02)	-0.05 (-0.11, 0.02)
20% least deprived		0.06 (-0.04, 0.16)	0.06 (-0.03, 0.16)	0.05 (-0.04, 0.14)		-0.02 (-0.11, 0.08)	-0.03 (-0.11, 0.06)	-0.03 (-0.12, 0.05)
CCE*IMD								
CCE*20% most deprived		0.02 (-0.01, 0.05)	0.02 (-0.01, 0.05)	0.03 (0.00, 0.05)*		0.01 (-0.01, 0.04)	0.02 (-0.00, 0.05)	0.02 (-0.01, 0.04)
CCE*20% least deprived		-0.02 (-0.06, 0.01)	-0.03 (-0.06, 0.01)	-0.02 (-0.05, 0.01)		0.01 (-0.02, 0.05)	0.02 (-0.02, 0.05)	0.02 (-0.01, 0.05)
Constant	-0.37 (-0.41, -0.34)***	-0.36 (-0.41, -0.31)***	0.34 (0.26, 0.42)***	0.09 (-0.14, 0.31)	-0.26 (-0.29, -0.23)***	-0.25 (-0.19, -0.21)***	0.53 (0.45, 0.60)***	0.16 (-0.07, 0.38)
F-statistics	724.63	146.90	85.19		482.61	97.13	79.74	85.30
Prob > F	0.000	0.000	0.000	0.000	0.000	0.000	0.000	0.000

For peer review only

Competing interests

The authors have no conflicts of interest to disclose.

Figure legend

Fig.1 Longitudinal association between cultural engagement and life satisfaction by levels of area deprivation (IMD 20% threshold). Data: Understanding Society, Waves 2 & 5. Estimates are derived from an OLS regression model including an interaction term (cultural engagement x levels of area deprivation).

Fig.2 Longitudinal association between cultural engagement and mental health functioning by levels of area deprivation (IMD 20% threshold). Data: Understanding Society, Waves 2 & 5. Estimates are derived from an OLS regression model including an interaction term (cultural engagement x levels of area deprivation).

References

1 Grossi E, Tavano GT, Sacco PL. Magic moments: Determinants of stress relief and subjective wellbeing from visiting a cultural heritage site. *Cult Med Psychiatry* 2019;**43**:4–24. doi:10.1007/s11013-018-9593-8

2 Morse N, Thomson LJM, Brown Z, *et al.* Effects of creative museum outreach sessions on measures of confidence, sociability and well-being for mental health and addiction recovery service- users. *Arts Health* 2015;**7**:231–46. doi:10.1080/17533015.2015.1061570

3 Wang S, Mak HW, Fancourt D. Arts, mental distress, mental health functioning & life satisfaction: Fixed-effects analyses of a nationally-representative panel study. *BMC Public Health* 2020;**20**:208. doi:10.1186/s12889-019-8109-y

4 Chatterjee HJ, Noble G. *Museums, Health and Wellbeing*. Farnham, UK; Burlington USA: : Ashgate Publishing Ltd 2013. doi:10.1080/13607863.2013.818101

5 Todd C, Camic PM, Lockyer B, *et al.* Museum-based programs for socially isolated older adults: Understanding what works. *Heal Place* 2017;**48**:47–55. doi:10.1016/j.healthplace.2017.08.005

6 Power A, Smyth K. Heritage, health and place: The legacies of local community-based heritage conservation on social wellbeing. *Heal Place* 2016;**39**:160–7. doi:10.1016/j.healthplace.2016.04.005

7 Bygren LO, Johansson S-E, Konlaan BB, *et al.* Attending cultural events and cancer mortality: A Swedish cohort study. *Arts Health* 2009;**1**:64–73. doi:10.1080/17533010802528058

8 Fancourt D, Steptoe A. The art of life and death: 14 year follow-up analyses of associations between arts engagement and mortality in the English Longitudinal Study of Ageing. *BMJ* 2009;**367**. doi:10.1136/bmj.l6377

9 Roberts S, Camic PM, Springham N. New roles for art galleries: Art-viewing as a community intervention for family carers of people with mental health problems. *Arts Health* 2011;**3**:146–59. doi:10.1080/17533015.2011.561360

10 Shaer D, Beaven K, Springham N, *et al.* The role of art therapy in a pilot for art-based Information Prescriptions at Tate Britain. *Int J Art Ther - Former Inscape* 2008;**13**:25–33. doi:10.1080/17454830802069877

For peer review only

11 Lackoi K, Patsou M, Chatterjee HJ et al. Museums for Health and Wellbeing. A Preliminary Report, National Alliance for Museums, Health and Wellbeing. 2016. https://museumsandwellbeingalliance.wordpress.com (accessed 6 Aug 2020).

12 Brook O. Spatial equity and cultural participation: how access influences attendance at museums and galleries in London. *Cult Trends* 2016;**25**:21–34.

13 Mak HW, Coulter R, Fancourt D. Does arts and cultural engagement vary geographically? Evidence from the UK Household Longitudinal Study. *Public Health* 2020;**185**:119–26.

14 Mak HW, Coulter R, Fancourt D. Is area deprivation associated with differential engagement in arts, culture and heritage? Evidence from three nationally-representative samples.

15 2015 English IMD explorer. http://dclgapps.communities.gov.uk/imd/idmap.html (accessed 26 Feb 2020).

16 Map of Museums in Britain. https://www.historic-uk.com/HistoryMagazine/DestinationsUK/Museums/ (accessed 1 May 2020).

17 Centre UWH. United Kingdom of Great Britain and Northern Ireland. UNESCO Wiorld Herit. Cent. 2019.https://whc.unesco.org/en/statesparties/gb (accessed 28 Oct 2019).

18 Widdop P, Cutts D. Cultural Trends Impact of place on museum participation. *Cult Trends* 2012;**21**:47–66. doi:10.1080/09548963.2012.641775

19 Houston D, Ong P. Arts accessibility to major museums and cultural/ethnic institutions in Los Angeles: can school tours overcome neighborhood disparities? *Environ Plan A* 2013;**45**:728–48. doi:10.1068/a45206

20 Bourdieu P. *Distinction: a social critique of the judgement of taste*. USA: Cambridge, Massachusetts: : Harvard University Press 1984. doi:10.1192/bjp.112.483.211-a

21 Duncan DT, Kawachi I. *Neighborhoods and health*. 2nd ed. New York: Oxford University Press 2018.

22 Adams RJ, Howard N, Tucker G, *et al.* Effects of area deprivation on health risks and outcomes: a multilevel, cross-sectional, Australian population study. *Int J Public Heal* 2009;**54**:183–92. doi:10.1007/s00038-009-7113-x

23 Macdonald L, Cummins S, Macintyre S. Neighbourhood fast food environment and area deprivation-substitution or concentration? *Appetite* 2007;**49**:251–4. doi:10.1016/j.appet.2006.11.004

24 Stafford M, Marmot M. Neighbourhood deprivation and health: Does it affect us all equally? *Int J Epidemiol* 2003;**32**:357–66. doi:10.1093/ije/dyg084

25 Smith SJ, Easterlow D. The strange geography of health inequalities. *Transitions of the Institute of British Geographers, New Series 2005; 30(2): 173-190.*

26 Smith T, Noble M, Noble S, *et al.* The English Indices of Deprivation 2015 Technical report. 2015. https://assets.publishing.service.gov.uk/government/uploads/system/uploads/attachment_data/file/464485/English_Indices_of_Deprivation_2015_-_Technical-Report.pdf (accessed 14 Aug 2020).

27 Drinkwater C, Wildman J, Moffatt S. Social prescribing. *Perfus* 2018;**364**:1–5. doi:10.1136/bmj.l1285

For peer review only

28 Hilary Bungay SRR A, Clift SB, of Health P. Arts on Prescription: A review of practice in the UK. *Perspect Public Health* 2010;**130**. doi:10.1177/1757913910384050

29 The Social Prescribing Network. Soc. Prescr. Netw. 2018.https://www.socialprescribingnetwork.com/ (accessed 28 Oct 2019).

30 Alderwick HAJ, Gottlieb LM, Fichtenberg CM, *et al.* Social Prescribing in the U.S. and England: Emerging Interventions to Address Patients' Social Needs. *Am J Prev Med* 2018;**54**:715–8. doi:10.1016/j.amepre.2018.01.039

31 Jensen A, Stickley T, Torrissen W, *et al.* Arts on prescription in Scandinavia: a review of current practice and future possibilities. *Perspect Public Health* 2017;**137**:268–74. doi:10.1177/1757913916676853

32 England AC. Creative People and Places. https://www.artscouncil.org.uk/get-funding/creative-people-and-places (accessed 28 Oct 2019).

33 Buck N, Mcfall S. Understanding Society: design overview. *Longit Life Course Stud* 2012;**3**:5–17.

34 Ware J, Kosinksi M, Keller S. A 12-Item Short-Form Health Survey: Construction of Scales and Preliminary Tests of Reliability and Validity. *Med Care* 1996;**34**:220–33. doi:10.1097/01.mlr.0000114910.46921.73

35 Kaminska O, Lynn P. Weighting and Sample Representation: Frequently Asked Questions. *Understanding Society: The UK Household Longitudinal Study* 2019.

36 Mak HW, Coulter R, Fancourt D. Patterns of social inequality in arts and cultural participation: findings from a nationally-representative sample of adults living in the UK. *WHO Public Heal Panor* 2020;**6**:55–68.

37 Bellis MA, Lowey H, Hughes K, *et al.* Variations in risk and protective factors for life satisfaction and mental wellbeing with deprivation: A cross-sectional study. *BMC Public Health* 2012;**12**:1–17. doi:10.1186/1471-2458-12-492

For peer review only

For peer review only

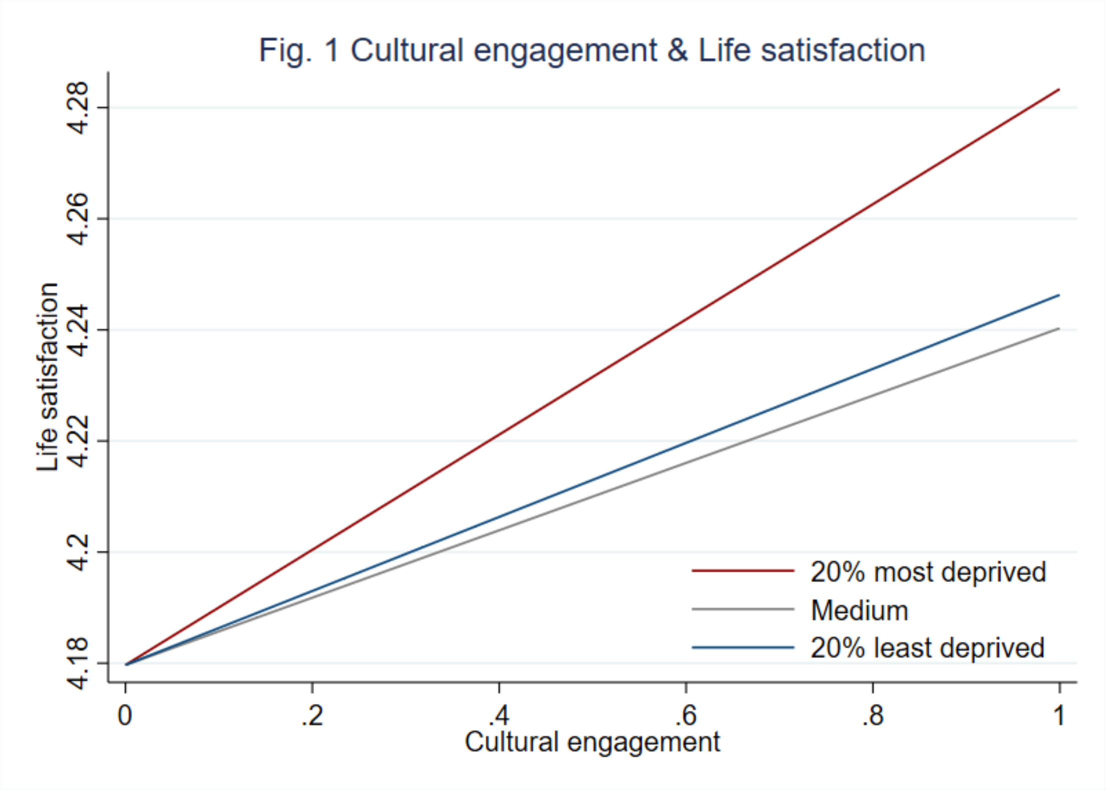

For peer review only

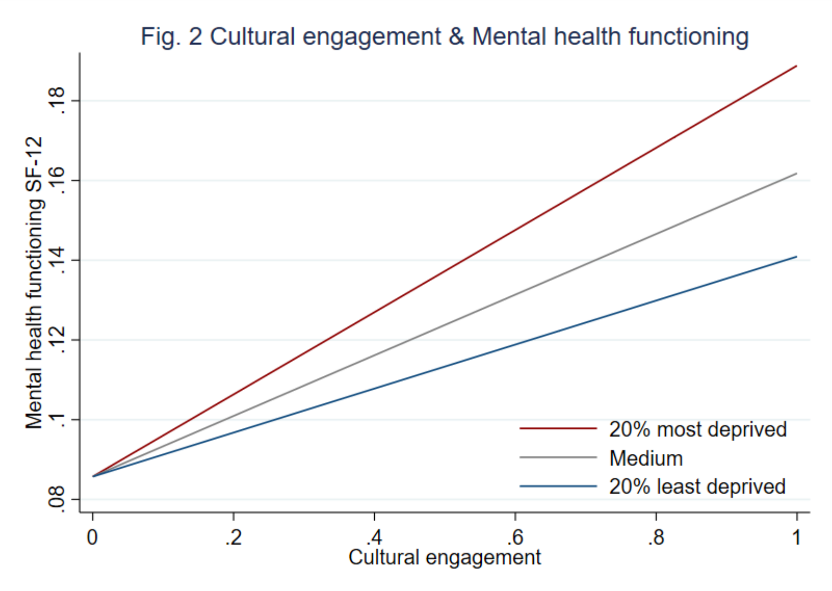

peer review only

Appendix

Cultural events

- A film at a cinema or other venue
- Exhibition or collection or art, photography or sculpture or a craft exhibition (not craft market)
- Event which included video or electronic art
- Event connected with books or writing
- Street arts or a public art display or installation (art in everyday surroundings, or an art work such as sculpture that is outdoors or in a public place)
- Carnival or culturally specific festival (for example, Mela, Baisakhi, Navrati, Feis)
- Circus (not animals)
- Play/drama, pantomime or a musical
- Opera/operetta
- Classical music performance
- Rock, pop or jazz performance
- Ballet
- Contemporary dance
- African people's dance or south Asian and Chinese dance

For peer review only

BMJ Open

Supplementary

Community cultural engagement and mental wellbeing at Wave 5 (IMD 10% threshold)

Table S1: OLS regression estimating the association between community cultural engagement (CCE) and mental wellbeing at Wave 5 (weighted; N=17,948)

	Cultural events				Museums & heritage sites			
	Basic model	+ IMD, interaction terms	+ Demographic factors	+ Socio-economic positions	Basic model	+ IMD, interaction terms	+ Demographic factors	+ Socio-economic positions
Life satisfaction								
CCE	0.10 (0.08, 0.12)***	0.10 (0.08, 0.12)***	0.11 (0.09, 0.13)***	0.07 (0.05, 0.09)***	0.11 (0.09, 0.12)***	0.11 (0.09, 0.13)***	0.09 (0.08, 0.11)***	0.05 (0.03, 0.08)***
IMD (ref: medium)								
10% most deprived		-0.13 (-0.26, 0.01)	-0.14 (-0.18, -0.01)*	-0.14 (-0.27, -0.01)*		-0.06 (-0.18, 0.06)	-0.07 (-0.19, 0.06)	-0.06 (-0.18, 0.06)
10% least deprived		0.13 (-0.06, 0.33)	0.13 (-0.06, 0.33)	0.13 (-0.07, 0.32)		0.16 (-0.02, 0.33)	0.15 (-0.03, 0.33)	0.16 (-0.01, 0.33)
CCE*IMD								
CCE*10% most deprived		0.05 (-0.00, 0.10)	0.05 (0.00, 0.11)*	0.05 (0.00, 0.11)*		0.02 (-0.03, 0.07)	0.02 (-0.03, 0.07)	0.02 (-0.03, 0.07)
CCE*10% least deprived		-0.03 (-0.10, 0.05)	-0.03 (-0.11, 0.05)	-0.03 (-0.11, 0.05)		-0.04 (-0.11, 0.02)	-0.05 (-0.12, 0.02)	-0.05 (-0.12, 0.02)
Constant	4.81 (4.76, 4.86)***	4.82 (4.77, 4.87)***	4.61 (4.48, 4.73)***	4.16 (3.80, 4.52)***	4.83 (4.78, 4.87)***	4.83 (4.78, 4.88)***	4.77 (4.65, 4.89)***	4.23 (3.87, 4.58)***
F-statistics	121.43	25.56	21.15	21.26	151.26	31.41	17.78	20.12
Prob > F	0.000	0.000	0.000	0.000	0.000	0.000	0.000	0.000
Mental health distress (GHQ-12)								
CCE	-0.06 (-0.07, -0.05)***	-0.06 (-0.07, -0.04)***	-0.06 (-0.08, -0.05)***	-0.04 (-0.06, -0.03)***	-0.05 (-0.06, -0.04)***	-0.05 (-0.06, -0.04)***	-0.05 (-0.06, -0.03)***	-0.03 (-0.04, -0.02)***
IMD (ref: medium)								
10% most deprived		0.08 (-0.01, 0.17)	0.08 (-0.01, 0.17)	0.08 (-0.01, 0.17)		0.04 (-0.04, 0.13)	0.04 (-0.04, 0.13)	0.04 (-0.04, 0.12)
10% least deprived		-0.09 (-0.21, 0.03)	-0.10 (-0.22, 0.02)	-0.10 (-0.22, 0.02)		-0.02 (-0.14, 0.10)	-0.03 (-0.15, 0.09)	-0.04 (-0.15, 0.08)
CCE*IMD								
CCE*10% most deprived		-0.03 (-0.06, 0.00)	-0.03 (-0.06, 0.00)	-0.03 (-0.06, 0.00)		-0.01 (-0.05, 0.01)	-0.01 (-0.05, 0.02)	-0.01 (-0.04, 0.02)
CCE*10% least deprived		0.03 (-0.02, 0.07)	0.03 (-0.02, 0.07)	0.03 (-0.02, 0.07)		-0.01 (-0.05, 0.04)	-0.01 (-0.05, 0.04)	-0.00 (-0.05, 0.04)
Constant	0.13 (0.10, 0.16)***	0.12 (0.09, 0.16)***	0.15 (0.07, 0.23)***	0.37 (0.15, 0.60)**	0.10 (0.07, 0.13)***	0.10 (0.06, 0.13)***	0.04 (-0.04, 0.12)	0.33 (0.10, 0.55)**
F-statistics	100.92	21.25	19.45	16.00	89.02	18.43	16.55	15.22
Prob > F	0.000	0.000	0.000	0.000	0.000	0.000	0.000	0.000
Mental health functioning SF-12								

For peer review only

CCE	0.16 (0.15, 0.18)***	0.17 (0.15, 0.18)***	0.14 (0.13, 0.15)***	0.08 (0.07, 0.09)***	0.13 (0.12, 0.14)***	0.13 (0.12, 0.14)***	0.12 (0.11, 0.13)***	0.07 (0.06, 0.09)***
IMD (ref: medium)								
10% most deprived		-0.04 (-0.14, 0.05)	-0.06 (-0.15, 0.04)	-0.06 (-0.15, 0.03)		-0.05 (-0.13, 0.04)	-0.05 (-0.13, 0.03)	-0.04 (-0.12, 0.04)
10% least deprived		0.10 (-0.03, 0.23)	0.10 (-0.03, 0.22)	0.10 (-0.02, 0.22)		0.02 (-0.10, 0.14)	0.01 (-0.10, 0.13)	0.03 (-0.09, 0.14)
CCE*IMD								
CCE*10% most deprived		0.01 (-0.03, 0.04)	0.01 (-0.02, 0.05)	0.01 (-0.02, 0.05)		0.01 (-0.02, 0.04)	0.01 (-0.02, 0.04)	0.01 (-0.02, 0.04)
CCE*10% least deprived		-0.03 (-0.08, 0.02)	-0.03 (-0.08, 0.01)	-0.03 (-0.08, 0.01)		0.01 (-0.04, 0.05)	0.01 (-0.04, 0.05)	-0.00 (-0.04, 0.04)
Constant	-0.37 (-0.41, -0.34)***	-0.37 (-0.41, -0.33)***	0.33 (0.25, 0.41)***	0.08 (-0.14, 0.30)	-0.26 (-0.29, -0.23)***	-0.26 (-0.29, -0.22)***	0.51 (0.44, 0.58)***	0.14 (-0.08, 0.37)
F-statistics	724.63	145.78	85.06	84.87	482.61	98.05	79.62	85.01
Prob > F	0.000	0.000	0.000	0.000	0.000	0.000	0.000	0.000

For peer review only

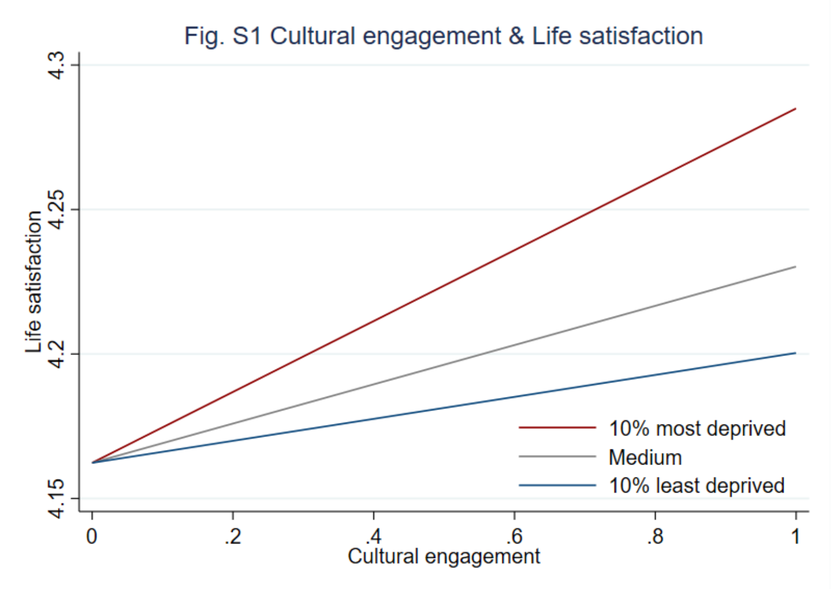

Fig. S1 Longitudinal association between cultural engagement and life satisfaction by levels of area deprivation (IMD 10% threshold). Data: Understanding Society, Waves 2 & 5. Estimates are derived from an OLS regression model including an interaction term (cultural engagement x levels of area deprivation).

For peer review only

Community cultural engagement and mental wellbeing at Wave 2

Table S2: OLS regression estimating the association between community cultural engagement (CCE) and life satisfaction at Wave 2 (weighted; N=32,861)

	Cultural events				Museums & heritage sites			
	Basic model	+ IMD, interaction terms	+ Demographic factors	+ Socio-economic positions	Basic model	+ IMD, interaction terms	+ Demographic factors	+ Socio-economic positions
IMD as a continuous variable								
CCE	0.11 (0.10, 0.12)***	0.12 (0.10, 0.14)***	0.14 (0.12, 0.17)***	0.11 (0.08, 0.13)***	0.11 (0.10, 0.13)***	0.12 (0.10, 0.15)***	0.11 (0.09, 0.14)***	0.08 (0.06, 0.11)***
IMD		0.01 (-0.00, 0.02)	0.01 (-0.00, 0.02)	0.01 (-0.00, 0.02)		0.01 (-0.00, 0.02)	0.01 (-0.00, 0.02)	0.01 (-0.00, 0.02)
CCE*IMD		-0.00 (-0.01, 0.00)	-0.00 (-0.01, 0.00)	-0.00 (-0.01, 0.00)		-0.00 (-0.01, 0.00)	-0.00 (-0.01, 0.00)	-0.00 (-0.01, 0.00)
Constant	4.99 (4.95, 5.02)***	4.94 (4.87, 5.00)***	4.62 (4.51, 4.72)***	3.81 (3.55, 4.07)***	5.02 (4.99, 5.05)***	4.97 (4.91, 5.03)***	4.82 (4.73, 4.92)***	3.92 (3.66, 4.18)***
F-statistics	328.32	111.06	61.43	52.60	352.61	118.47	54.25	49.83
Prob > F	0.000	0.000	0.000	0.000	0.000	0.000	0.000	0.000
Using 20% thresholds								
CCE	0.11 (0.10, 0.12)***	0.12 (0.11, 0.14)***	0.14 (0.13, 0.16)***	0.11 (0.09, 0.12)***	0.11 (0.10, 0.13)***	0.12 (0.10, 0.13)***	0.11 (0.09, 0.13)***	0.08 (0.06, 0.09)***
IMD (ref: medium)								
20% most deprived		0.05 (-0.03, 0.12)	0.03 (-0.04, 0.11)	0.04 (-0.04, 0.11)		0.02 (-0.05, 0.09)	0.01 (-0.06, 0.08)	0.01 (-.005, 0.08)
20% least deprived		0.16 (0.06, 0.25)**	0.15 (0.06, 0.25)**	0.15 (0.06, 0.25)**		0.11 (0.03, 0.20)**	0.11 (0.02, 0.19)*	0.11 (0.03, 0.20)**
CCE*IMD								
CCE*20% most deprived		-0.02 (-0.05, 0.01)	-0.02 (-0.05, 0.01)	-0.02 (-0.05, 0.01)		-0.01 (-0.04, 0.02)	-0.01 (-0.03, 0.02)	-0.01 (-0.03, 0.02)
CCE*20% least deprived		-0.04 (-0.07, -0.00)*	-0.04 (-0.08, -0.01)*	-0.04 (-0.07, -0.01)*		-0.02 (-0.06, 0.01)	-0.02 (-0.06, 0.01)	-0.02 (-0.06, 0.01)
Constant	4.99 (4.95, 5.02)***	4.95 (4.90, 4.99)***	4.63 (4.54, 4.73)***	3.83 (3.57, 4.09)***	5.02 (4.99, 5.05)***	5.00 (4.96, 5.04)***	4.85 (4.77, 4.94)***	3.95 (3.69, 4.21)***
F-statistics	328.32	67.78	53.99	48.64	352.61	71.92	47.76	46.08
Prob > F	0.000	0.000	0.000	0.000	0.000	0.000	0.000	0.000

For peer review only

Table S3: OLS regression estimating the association between community cultural engagement (CCE) and mental health distress (GHQ-12) at Wave 2 (weighted; N=32,861)

	Cultural events				Museums & heritage sites			
	Basic model	+ IMD, interaction terms	+ Demographic factors	+ Socio-economic positions	Basic model	+ IMD, interaction terms	+ Demographic factors	+ Socio-economic positions
IMD as a continuous variable								
CCE	-0.08 (-0.09, -0.07)***	-0.09 (-0.11, -0.08)***	-0.09 (-0.11, -0.08)***	-0.07 (-0.09, -0.06)***	-0.06 (-0.07, -0.06)***	-0.07 (-0.09, -0.05)***	-0.07 (-0.08, -0.05)***	-0.05 (-0.06, -0.03)***
IMD		-0.01 (-0.02, -0.00)*	-0.01 (-0.02, -0.00)*	-0.01 (-0.02, -0.00)*		-0.01 (-0.01, 0.00)	-0.01 (-0.01, 0.00)	-0.01 (-0.01, 0.00)
CCE*IMD		0.00 (-0.00, 0.01)	0.00 (-0.00, 0.01)	0.00 (-0.00, 0.01)		0.00 (-0.00, 0.00)	0.00 (-0.00, 0.00)	0.00 (-0.00, 0.00)
Constant	0.16 (0.14, 0.18)***	0.21 (0.16, 0.25)***	-0.11 (0.05, 0.18)**	0.51 (0.33, 0.69)***	0.10 (0.08, 0.12)***	0.13 (0.09, 0.17)***	-0.03 (-0.10, 0.03)	0.43 (0.25, 0.61)***
F-statistics	389.38	130.71	51.46	41.63	247.47	83.24	41.45	38.40
Prob > F	0.000	0.000	0.000	0.000	0.000	0.000	0.000	0.000
Using 20% thresholds								
CCE	-0.08 (-0.09, -0.07)***	-0.08 (-0.09, -0.07)***	-0.08 (-0.09, -0.07)***	-0.06 (-0.07, -0.05)***	-0.06 (-0.07, -0.06)***	-0.07 (-0.08, -0.05)***	-0.06 (-0.07, -0.05)***	-0.04 (-0.06, -0.03)***
IMD (ref: medium)								
20% most deprived		0.02 (-0.03, 0.07)	0.03 (-0.02, 0.08)	0.02 (-0.03, 0.08)		-0.01 (-0.05, 0.04)	-0.01 (-0.05, 0.04)	-0.01 (-0.05, 0.04)
20% least deprived		-0.04 (-0.11, 0.02)	-0.04 (-0.11, 0.02)	-0.04 (-0.11, 0.02)		-0.04 (-0.10, 0.02)	-0.04 (-0.10, 0.01)	-0.05 (-0.10, 0.01)
CCE*IMD								
CCE*20% most deprived		-0.01 (-0.03, 0.01)	-0.01 (-0.03, 0.01)	-0.01 (-0.03, 0.01)		0.01 (-0.01, 0.02)	0.01 (-0.01, 0.02)	0.01 (-0.01, 0.02)
CCE*20% least deprived		0.00 (-0.02, 0.03)	0.00 (-0.02, 0.03)	0.00 (-0.02, 0.03)		0.00 (-0.02, 0.03)	0.01 (-0.02, 0.03)	0.01 (-0.02, 0.03)
Constant	0.16 (0.14, 0.18)***	0.16 (0.13, 0.19)***	0.07 (0.01, 0.13)*	0.47 (0.29, 0.65)***	0.10 (0.08, 0.12)***	0.11 (0.08, 0.14)***	-0.06 (-0.11, 0.00)	-0.41 (0.23, 0.59)***
F-statistics	389.38	79.01	45.28	38.47	247.47	50.09	36.40	35.37
Prob > F	0.000	0.000	0.000	0.000	0.000	0.000	0.000	0.000

For peer review only

For peer review only - http://bmjopen.bmj.com/site/about/guidelines.xhtml

Table S4: OLS regression estimating the association between community cultural engagement (CCE) and mental health functioning (SF-12) at Wave 2 (weighted; N=32,861)

	Cultural events				Museums & heritage sites			
	Basic model	+ IMD, interaction terms	+ Demographic factors	+ Socio-economic positions	Basic model	+ IMD, interaction terms	+ Demographic factors	+ Socio-economic positions
IMD as a continuous variable								
CCE	0.19 (0.18, 0.20)***	0.21 (0.19, 0.22)***	0.17 (0.15, 0.18)***	0.11 (0.10, 0.13)***	0.14 (0.13, 0.15)***	0.15 (0.13, 0.16)***	0.13 (0.12, 0.15)***	0.08 (0.07, 0.10)***
IMD		0.01 (0.00, 0.02)**	0.01 (0.00, 0.02)**	0.01 (0.00, 0.02)**		0.01 (0.00, 0.01)*	0.01 (0.00, 0.01)*	0.01 (0.00, 0.01)
CCE*IMD		-0.00 (-0.01, -0.00)**	-0.00 (-0.01, -0.00)**	-0.00 (-0.01, -0.00)**		-0.00 (-0.00, 0.00)	-0.00 (-0.00, 0.00)	-0.00 (-0.00, 0.00)
Constant	-0.37 (-0.39, -0.35)***	-0.43 (-0.47, -0.38)***	0.40 (0.33, 0.46)***	0.14 (-0.02, 0.30)	-0.22 (-0.24, -0.20)***	-0.26 (-0.30, -0.22)***	0.64 (0.58, 0.69)***	0.26 (0.10, 0.41)**
F-statistics	2132.48	715.63	285.11	245.22	1214.98	407.30	259.00	240.24
Prob > F	0.000	0.000	0.000	0.000	0.000	0.000	0.000	0.000
Using 20% thresholds								
CCE	0.19 (0.18, 0.20)***	0.19 (0.17, 0.20)***	0.15 (0.14, 0.16)***	0.09 (0.08, 0.10)***	0.14 (0.13, 0.15)***	0.14 (0.13, 0.15)***	0.12 (0.11, 0.13)***	0.08 (0.07, 0.09)***
IMD (ref: medium)								
20% most deprived		-0.05 (-0.10, 0.00)	-0.04 (-0.09, 0.01)	-0.04 (-0.09, 0.00)		-0.02 (-0.07, 0.03)	-0.02 (-0.06, 0.03)	-0.01 (-0.05, 0.03)
20% least deprived		0.07 (0.00, 0.14)*	0.07 (0.01, 0.14)*	0.07 (0.00, 0.13)*		0.04 (-0.02, 0.10)	0.04 (-0.01, 0.10)	0.04 (-0.01, 0.09)
CCE*IMD								
CCE*20% most deprived		0.02 (0.00, 0.04)*	0.02 (0.00, 0.04)*	0.02 (0.00, 0.04)*		0.01 (-0.01, 0.02)	0.01 (-0.01, 0.02)	0.00 (-0.01, 0.02)
CCE*20% least deprived		-0.01 (-0.04, 0.01)	-0.02 (-0.04, 0.01)	-0.01 (-0.04, 0.01)		-0.00 (-0.02, 0.02)	-0.00 (-0.02, 0.02)	-0.00 (-0.02, 0.02)
Constant	-0.37 (-0.39, -0.35)***	-0.36 (-0.40, -0.33)***	0.46 (0.40, 0.51)***	0.19 (0.04, 0.35)*	-0.22 (-0.24, -0.20)***	-0.22 (-0.25, -0.19)***	0.67 (0.62, 0.72)***	0.28 (0.13, 0.44)***
F-statistics	2132.48	430.12	250.66	226.44	1214.98	243.98	227.44	221.50
Prob > F	0.000	0.000	0.000	0.000	0.000	0.000	0.000	0.000

For peer review only

BMJ Open

Associations between community cultural engagement and life satisfaction, mental distress and mental health functioning using data from the UK Household Longitudinal Study (UKHLS): are associations moderated by area deprivation?

Journal:	*BMJ Open*
Manuscript ID	bmjopen-2020-045512.R1
Article Type:	Original research
Date Submitted by the Author:	13-Jul-2021
Complete List of Authors:	Mak, Hei Wan; University College London, Department of Behavioural Science and Health<br>Coulter, Rory; University College London, Department of Geography<br>Fancourt, Daisy; University College London Research Department of Epidemiology and Public Health, Department of Behavioural Science and Health
**Primary Subject Heading**:	Public health
Secondary Subject Heading:	Public health
Keywords:	MENTAL HEALTH, PUBLIC HEALTH, SOCIAL MEDICINE

SCHOLARONE™ Manuscripts

I, the Submitting Author has the right to grant and does grant on behalf of all authors of the Work (as defined in the below author licence), an exclusive licence and/or a non-exclusive licence for contributions from authors who are: i) UK Crown employees; ii) where BMJ has agreed a CC-BY licence shall apply, and/or iii) in accordance with the terms applicable for US Federal Government officers or employees acting as part of their official duties; on a worldwide, perpetual, irrevocable, royalty-free basis to BMJ Publishing Group Ltd ("BMJ") its licensees and where the relevant Journal is co-owned by BMJ to the co-owners of the Journal, to publish the Work in this journal and any other BMJ products and to exploit all rights, as set out in our licence.

The Submitting Author accepts and understands that any supply made under these terms is made by BMJ to the Submitting Author unless you are acting as an employee on behalf of your employer or a postgraduate student of an affiliated institution which is paying any applicable article publishing charge ("APC") for Open Access articles. Where the Submitting Author wishes to make the Work available on an Open Access basis (and intends to pay the relevant APC), the terms of reuse of such Open Access shall be governed by a Creative Commons licence – details of these licences and which Creative Commons licence will apply to this Work are set out in our licence referred to above.

Other than as permitted in any relevant BMJ Author's Self Archiving Policies, I confirm this Work has not been accepted for publication elsewhere, is not being considered for publication elsewhere and does not duplicate material already published. I confirm all authors consent to publication of this Work and authorise the granting of this licence.

For peer review only

Associations between community cultural engagement and life satisfaction, mental distress and mental health functioning using data from the UK Household Longitudinal Study (UKHLS): are associations moderated by area deprivation?

Hei Wan Mak[1], Rory Coulter[2], Daisy Fancourt[1]

[1]Department of Behavioural Science and Health, University College London, London, UK

[2]Department of Geography, University College London, London, UK

Corresponding author: Daisy Fancourt (d.fancourt@ucl.ac.uk)

Keywords: community engagement; area deprivation; life satisfaction; mental distress; mental health functioning

Word count: 3922

For peer review only

Abstract

Objectives. The association between community cultural engagement and mental health and wellbeing is well-established. However, little is known about whether such associations are influenced by area characteristics. This study therefore examined whether the association between engagement in community cultural assets (attendance at cultural events; visiting museums and heritage sites) and subsequent wellbeing (life satisfaction, mental distress and mental health functioning) is moderated by neighbourhood deprivation.

Design. Data were drawn from Understanding Society: The UK Household Longitudinal Study (UKHLS) Waves 2 and 5. Participating households' addresses were geocoded into statistical neighbourhood zones categorised according to their level of area deprivation.

Setting. General population.

Participants. UK general adult population, with a total sample of 14,783.

Main outcome measures. Life satisfaction was measured with a 7-point scale (1-completely unsatisfied to 7-completely satisfied). Mental distress was measured using the GHQ-12 (General Health Questionnaire). Mental health functioning was measured using SF-12 (12-Item Short Form Health Survey).

Results. Using OLS regression, we found that engagement in cultural assets was consistently and positively associated with subsequent life satisfaction and mental health functioning and negatively associated with mental distress. Importantly, such associations were independent of individuals' demographic background, socio-economic characteristics and regional location. The results also show that relationships between engagement in community cultural assets and wellbeing were stronger in more deprived areas.

Conclusions. This study shows that engagement in community cultural assets is associated with better wellbeing, with some evidence that individuals in areas of high deprivation potentially may benefit more from these engagements. Given that causal mechanisms were

For peer review only

not tested, causal claims cannot be generated from the results. However, the results suggest that place-based funding schemes that involve investment in areas of higher deprivation to improve engagement rates should be explored further to see if they can help promote better wellbeing amongst residents.

Strengths and limitations of this study

- This is one of the first large, population-based study to investigate whether the association between community cultural engagement and mental wellbeing varies with local deprivation.
- This study was based on a nationally representative sample with a 3-year follow up period and the analysis controlled for a wide range of important confounding factors.
- However, as this was an observational study, causality cannot be conclusively established.
- The Index of Multiple Deprivation is comprised of various weighted components (e.g. living environment, income and employment deprivation) which combine to create a proxy of neighbourhood deprivation.

Ethics approval

The University of Essex Ethics Committee has approved all data collection on Understanding Society: The UK Household Longitudinal Study (UKHLS) main study and innovation panel waves, including asking consent for all data linkages except to health records. Respondents aged 16 or above provided written consent to participate.

Availability of data and materials

UKHLS data is available from the UK Data Service https://discover.ukdataservice.ac.uk/catalogue/?sn=6614. Data documentation is available from the Understanding Society website https://www.understandingsociety.ac.uk/documentation. Deprivation data are captured through linking Lower Layer Super Output areas (LSOA) data from the UKHLS with data from the English Index of Multiple Deprivation (IMD 2015).

For peer review only

Funding

DF is supported by the Wellcome Trust [205407/Z/16/Z]. HWM is funded through the AHRC project HEARTS [AH/P005888/1]. This project is also supported by UCL Grand Challenges Award "Environment and Wellbeing" [551987/100/156425 33002] and ESRC WELLCOMM project [ES/T006994/1].

Author Contributions

HWM conducted the data management, data analyses and provided input on the manuscript. RC and DF assisted with analytical issues, provided input on the analytical scheme and the manuscript. All authors are responsible for reported research and have participated in the concept and design, analysis and interpretation of data, and drafting and revising of the manuscript.

Introduction

The role of community cultural engagement (CCE) in health and wellbeing has received increased attention across a number of disciplines over the past two decades. CCE can

For peer review only

include going to cultural events (such as the theatre, opera, concerts or exhibitions) and visiting museums and heritage sites (including visiting a city or town with historic character), and has been associated with improvements in wellbeing [1–3], slower declines in cognition [4], reduced levels of isolation and loneliness [5]), enhanced social wellbeing in the community [6] and lower mortality rates [7,8]. These benefits have been found amongst healthy individuals as well as people with mental health problems, dementia, substance use addiction, carers, families living in deprived areas, asylum seekers and isolated adults [9–11].

Unfortunately, previous studies have suggested a social and geographical gradient in cultural engagement, in which the engagement rate is higher amongst people living in more affluent areas [12–14]. Certainly, CCE relies on cultural assets (i.e. tangible spaces, buildings or organisations) being available within communities, and most community cultural assets are unevenly distributed across the UK in ways that co-vary with deprivation [15–17]. In addition, even when cultural assets are available, the characteristics of neighbourhoods — such as the living environment, local facilities and services, social connections and networks, levels of accessibility, and the safety of the area — have been identified as specific factors that can affect the engagement rate [12,14,18,19]. As such some people may have greater, safer, and easier opportunities to engage with culture (the 'contextual effect'). But it is worth noting that this effect may be less obvious in those deprived parts of England that lie in close proximity to more affluent areas with greater availability of assets [15–17].

It has also been shown that, independent of area deprivation, people living in particular areas tend to cluster together according to their demographics (e.g. age, ethnicity), socio-economic position (ability to afford housing) and lifestyle or cultural preferences. This can lead to the acquisition of common norms, values, and economic and cultural capital, which may lead to different behavioural patterns of CCE within specific neighbourhoods (known as a 'compositional effect') [20,21]. Compositional effects have been demonstrated for CCE in previous studies. For instance, "cosmopolitan" or "student" areas of England have been identified as having particularly strong patterns of CCE relative to, say, post-industrial communities [13].

However, variations in people's CCE behaviours may not be simply a matter of contextual and compositional effects. It is plausible that the places people live is not only a *predictor* of CCE,

For peer review only

but also a *moderator* of the association between CCE and health outcomes. There is evidence of similar social and geographical gradients in CCE and health, with many studies showing spatially variable associations between area deprivation and poor health outcomes, although results have been complex and not entirely consistent [22–24]. Indeed, many studies examining health inequality have incorporated geographical data and have suggested that unequal access to health-promoting environments, such as the availability of and accessibility to cultural and artistic activities, could contribute to the spatial health divide [12,25]. This is particularly relevant to areas of deprivation where educational levels, employment rates and living standards are lower and mental health problems are more common [26]. Therefore, it is plausible that bringing cultural resources to deprived areas and improving community infrastructure could have a greater impact on people's wellbeing in these areas than in affluent areas, as in deprived areas there may otherwise be limited assets and opportunities to build positive mental health.

In light of this, it is relevant to explore whether place is important not just in predicting levels of CCE but also in moderating the relationship between CCE and health outcomes. Understanding whether there is any moderation is crucial and relevant to current public health strategies and interventions such as 'social prescribing' schemes and place-based funding streams for the cultural sector. These are predicated on the belief that increasing the local availability of assets and their usage could lead to increased CCE and thus improved health outcomes [27–32]. But it remains unclear whether investment in cultural assets in different locations holds equal potential for positively influencing health.

Therefore, in this paper, we used a large longitudinal and nationally-representative sample of adults to examine whether the association between engagement in community cultural assets and subsequent wellbeing (operationalised as life satisfaction, mental distress and mental health functioning) is moderated by geographical deprivation in the origin location. In particular, given that differential exposure to risks affects health differently, we explored whether individuals living in areas of high deprivation who are at higher risks of experiencing poorer mental health gain the same or even greater wellbeing benefits from CCE as individuals living in areas of low deprivation.

For peer review only

Data & Methods

We used data from Understanding Society: The UK Household Longitudinal Study (UKHLS), which is a continuation of the long-running British Household Panel Survey. UKHLS follows over 50,000 individuals from 30,000 households annually [33] and collects rich information on engagement in cultural events and museums and heritage. Crucially, the survey also collects a suite of measures of self-reported individual wellbeing. The University of Essex Ethics Committee approved the study and participants provided informed consent.

In order to investigate the role of area deprivation, we used geo-coded UKHLS data in which participating households' addresses have been positioned into a number of spatial zoning systems (e.g. administrative and census statistical geographies). For our analysis, we extracted a sample of adults living in England who responded to the Wave 2 (2010/12; response rate=84%), where data on engagement in CCE were first available, and Wave 5 (2013/15; response rate=85%) interviews. We only considered respondents who completed both Waves 2 and 5 interviews and those who answered across all measures, as well as respondents with a valid sampling weight value.

Patient and public involvement

This study used publicly available secondary data from the UK Data Service (https://www.ukdataservice.ac.uk/). Patients and the public were not involved.

Measures

We defined neighbourhoods as 2011 census Lower Layer Super Output Areas (LSOA) and matched the 2011 LSOA with Wave 2 UKHLS where data were collected between 2010/12. LSOAs are designed for the consistent reporting of small area statistics in England and Wales. Using the LSOA geocodes, we attached the 2015 English Index of Multiple Deprivation (IMD 2015), which measures the relative deprivation of small areas across seven domains: income, employment, health deprivation and disability, education, skills and training, crime, barriers to housing and service and living environment. Our main analysis was based on the IMD decile rank values which we used as a continuous measure.

For CCE, we focused on attendance at cultural events; including going to the theatre, concerts, opera and exhibition, and museums/galleries and heritage sites visits (a full list of CCE is

For peer review only

provided in Appendix I). CCE was measured in Wave 2. At this wave, respondents were asked how often they had attended any of the cultural events, visited museums/galleries and visited heritage sites in the past 12 months. Frequency of engagement with these activities was categorised as “not once in the last 12 months”, “once in the last 12 months”, “twice in the last 12 months”, “less often than once a month but at least 3 or 4 times a year”, “less often than once a week but at least once a month” and “at least once a week”. Due to the similar nature of the activities, visits in museums/galleries and heritage sites were collapsed into one variable. Both types of CCE (cultural attendance and museum and heritage engagement) were treated as continuous measures.

We explored three outcome wellbeing measures in Wave 5, which took place around three years after our Wave 2 baseline. These measures were life satisfaction, mental distress and mental health functioning. Life satisfaction was measured through asking respondents how satisfied they felt with their life overall, with responses ranging from 1 (completely unsatisfied) to 7 (completely satisfied). This measure has been used as one of the questions to measure personal wellbeing in the UK general population by the UK Office for National Statistics, albeit the scale varies slightly [34]. Mental distress was measured using the GHQ-12 (General Health Questionnaire); a screening device identifying psychiatric disorders in the general population and in primary medical care settings [35,36]. The GHQ-12 self-reported questionnaire includes 12 4-point items (such as sleeping problems, overall happiness and depressive symptoms; α=0.91). The scale was computed additively, ranging from 1 to 4; higher scores indicating a greater incidence of mental distress. Mental health functioning was measured using SF-12 (12-Item Short Form Health Survey; α=0.90); a well-validated survey that was designed to measure respondents’ general health-related quality of life. The 12-item survey contains eight indicators: physical functioning, role limitations due to physical health problems, bodily pain, general health, vitality, social functioning, role limitations due to emotional problems and mental health [37]. The scale was computed additively, ranging from 1 to 5; higher scores indicating better levels of mental health functioning.

In our analysis, we controlled for broad regional variations (North, Midlands, South) as well as demographic and socio-economic variables in Wave 2. Demographic control variables included age, gender (female vs male), ethnicity (White, Asian/Asian British, Black/Black British, mixed/others), partnership status (married/in cohabitation, single and never

For peer review only

married/never in civil partnership, separated/divorced/widowed), presence of child(ren) under the age of 16, and whether respondents were living alone. Socio-economic controls included educational level (university degree, advanced (A-levels) exams/higher education (e.g. a Higher Education Certificate/BTEC), passed General Certificate of Secondary Education-GCSE, unrecognised qualification/no qualification), occupational socio-economic status (managerial/professional, intermediate/small employment/own account, lower supervision/lower technician/semi-routine/routine, not in employment (e.g. unemployed or retired or students)), household monthly gross income (logged), and housing tenure (house owner, social rent, private rent).

Statistical analysis

To understand whether the relationship between CCE (x) and mental wellbeing measures (y) varied with area deprivation (the potential moderator), we ran a cross-sectional analysis using OLS regression models. Given that residential location is highly correlated with personal demographic and socio-economic factors, the regression models were constructed sequentially to understand the changes of the association between CCE and mental wellbeing. In Model 1 (basic model), we included only CCE. Model 2 additionally controlled for IMD and the interaction terms (i.e. CCE*IMD). Model 3 additionally included demographic factors, and finally Model 4 adjusted for socio-economic position. All models were weighted using inverse probability weights derived from the Wave 2 longitudinal weights supplied with UKHLS. These weights have been tailored to the analytical sample and should correct our estimates to take into account differential sample selection and retention probabilities [38]. List-wise deletion was used to handle small volumes of missing data (0.3%).

To check whether our data met the assumption of OLS regressions, we ran a series of regression diagnostic tests. Our tests show that the distribution of residuals was almost homoscedastic and normal for models estimating mental distress and mental health functioning. The distribution was less satisfactory for models estimating life satisfaction, most likely due to the more discrete scale of this variable. However, our large sample size and the fact we present weighted estimates with robust standard errors should mitigate against problems arising from model fit. Nonetheless, more caution should be exercised when interpreting the results of the life satisfaction models as compared with the other well-being

For peer review only

outcomes. The risk of multicollinearity was also very low with a mean Variance Inflation Factor (VIF) of 2.03 among the independent variables.

As further robustness checks, all analyses were replicated with two three-fold deprivation measures using 20% IMD threshold and 10% IMD threshold (i.e. 10%/20% most deprived areas, 10%/20% least deprived areas, and the remaining intermediate neighbourhoods). All analyses were carried out using Stata 16.

Results

In our sample, 38,069 participants living in England responded to the Wave 2 and 30,635 participants responded to the Wave 5 interviews. 25,464 individuals completed both interviews. Of these, 23,247 participants provided answers for the outcome variables, with 23,244 respondents additionally answered questions on CCE, and 22,463 individuals also answered across all other measures. Amongst them, 14,833 received a non-zero Wave 2 longitudinal weight value provided by UKHLS. Of these, all but 50 (14,783) received a valid tailored weight value for this analysis.

(Table 1)

Table 1 reports weighted descriptive statistics of the unweighted and weighted samples. In our weighted sample, the average age was 47 years, 52% were female, and over 90% were of white ethnicity. In relation to socio-economic position, 35% of the respondents had a university degree, 27% were in managerial/professional roles and 72% owned a house. On average, the scores of life satisfaction, mental health distress and mental health functioning were 5.1, 1.9, and 3.8 respectively. 22% reported of not attending any cultural events and 27% reported of not visiting museums or heritage sites in the past 12 months. Around 19% attended cultural events and 15% visited museums or heritage sites at least once a month.

(Tables 2-4)

Life satisfaction

After adjusting for individual demographic factors and socio-economic position, both types of CCE were associated with a slightly greater level of life satisfaction (attending cultural events: coef=0.08, 95%CI=0.04, 0.13, standardised beta=0.08; visiting museums & heritage sites: coef=0.06, 95%CI=0.02, 0.11, beta=0.06) (Table 2). There was no evidence that this

For peer review only

association was moderated by area deprivation when using the continuous measure, nor when using the three-fold 10% or 20% measure (Tables 2, STables 1 & 2).

Mental distress (GHQ-12)

When accounting for individual demographic and socio-economic characteristics, attending cultural events was associated with lower levels of mental distress (coef=-0.03, 95%CI=-0.04, -0.01, beta=-0.09) and so was visiting museums and heritage sites (coef=-0.02, 95%CI=-0.03, -0.00, beta=-0.05). There was some evidence of moderation by area deprivation shown in cultural attendance, although the moderation was attenuated when adjusting for socio-economic position (Table 3). To explore the interaction effects, we estimated marginal effects. When comparing with the most distinct cultural attendance frequency (i.e. at least once a week vs none in the past 12 months), the score of mental distress amongst people living in the 10% least deprived areas was decreased from 1.89 (no engagement) to 1.83 (weekly engagement). Similarly, of people living in the 10% most deprived areas, their mental distress score was decreased from 2.05 (no engagement) to 1.89 (weekly engagement). The differences in mental distress scores between people living in varying levels of area deprivation became smaller with increased cultural attendance frequency (Figure 1). No moderation association was found for museum and heritage engagement, nor when using the three-fold 20% IMD measure (STable 3) and three-fold 10% IMD measure (STable 4) to test for non-linear moderations.

Mental health functioning (SF-12)

After adjusting for demographic background and socio-economic position, both types of CCE were associated with a higher level of subsequent mental health functioning (attending cultural events: coef=0.06, 95%CI=0.04, 0.08, beta=0.13; visiting museums & heritage sites: coef=0.05, 95%CI=0.03, 0.06, beta=0.10). There was some indication of moderation of the association by area deprivation for both types of CCE, although the moderation was attenuated when adjusting for socio-economic position for cultural engagement and when adjusting for demographic factors for museum and heritage engagement (Table 4). Of those living in the 10% least deprived areas, their score in mental health functioning was increased from 3.79-3.80 (no engagement) to 4.07 (weekly engagement) for both types of CCE. Amongst those living in the 10% most deprived areas, their score was increased from 3.39-3.44 (no engagement) to 3.85-3.86 (weekly engagement). The differences in mental health functioning

For peer review only

scores between people living in varying levels of area deprivation became narrower with increased CCE (Figures 2 & 3). The moderation associations were also reflected when using the three-fold IMD measures to test for non-linearities, although they were less prominent with the 20% threshold (STables 5 & 6).

(Figures 1-3)

Sensitivity analyses

Additional sensitivity checks were conducted using outcome data from Wave 2 rather than Wave 5. In these checks, we estimated the same models as discussed above but this time relating Wave 2 CCE and IMD values to contemporaneously measured Wave 2 life satisfaction, mental distress and mental health functioning outcomes. The results were broadly in line with the findings discussed above and are available from the lead author on request.

Discussion

This is one of the first large population-based study to investigate whether the association between CCE (attending cultural events or visiting museums and heritages sites) and mental wellbeing (including life satisfaction, mental distress and mental health functioning) varies with local deprivation. In line with previous research, our results show that engagement in cultural assets was consistently and positively associated with subsequent life satisfaction and mental health functioning and negatively associated with mental distress [3]. Importantly, this paper found that such associations were independent of individuals' demographic background, socio-economic characteristics and regional locations. In particular, our models show that every one standard deviation increase in CCE is associated with higher life satisfaction and mental health functioning (by 0.06-0.13 standard deviations) and lower mental distress (by 0.05-0.09 standard deviations). Although the magnitude of these effects is fairly small, such associations were evident even after considering levels of area deprivation, demographics, and socio-economic factors and when predicting the outcomes measured after three years. This suggests that, while social and geographical factors can influence engagement rates [12–14,18,39], CCE is consistently associated with minor improvements in mental health and wellbeing regardless of where people live.

Notably, our analysis shows some indications of interactions between both types of CCE and area deprivation. In particular, we found that the rates of growth in mental health functioning

For peer review only

that accompany CCE are stronger amongst people living in deprived areas, and that the rates of decline in mental distress that accompany cultural events attendance are also more prominent amongst those living in deprived areas. This suggests that individuals who live in highly deprived areas may benefit the most from CCE in terms of mental health and wellbeing improvements. This provides an important insight by suggesting that when individuals living in deprived areas are offered the opportunities to engaging in CCE, they could potentially experience greater improvements in mental health and wellbeing than those living in wealthy areas who have already been able to benefit from higher levels of engagement (possibly since childhood) and who usually enjoy other social advantages (e.g. higher social positions) that fortify their mental wellbeing. In addition, the differences in mental wellbeing between people living in varying levels of deprivation become somewhat smaller as CCE increases. Despite this, deprivation appears to be a consistent barrier to engagement [13,14,18]. People living in deprived areas are less likely to engage in CCE not simply because of their personal background and characteristics (e.g. lower educational attainment and occupational position), but because of the areas they reside in offer less cultural opportunities (e.g. unsafe, culturally deprived, undesirable) [14,18]. Consequently, unequal access to the arts may inhibit people living in deprived communities from enjoying the benefits provided by CCE and hence exacerbate social, cultural and health inequalities. No moderations are found for life satisfaction, nor for engagement in museum and heritage and mental distress. This suggests that the benefits of CCE on life satisfaction and mental distress are similar regardless of residential locations.

The results of this study have clear implications for the design and roll-out of place-based programmes for CCE that operate under the assumption that investing in areas of high deprivation and low cultural opportunity could improve wellbeing levels. One example of such a scheme is the UK 'Creative People and Places' funded by Arts Council England. The data presented here suggest that such schemes could have the potential to achieve their aims as there is some indication that the benefits of CCE may be slightly more pronounced in more deprived areas. But there are mixed implications for other schemes that aim to signpost individuals to existing community cultural activities to improve health and wellbeing, as occurs in 'social prescribing'. While such an approach clearly has some potential to achieve its health aims in the same way as place-based funding schemes, social prescribing schemes rely on

For peer review only

the availability of community cultural assets within communities. It has been suggested that CCE can be increased in areas with more cultural opportunity structures and in areas with high accessibility to cultural infrastructures, especially for groups that have often been excluded from CCE [12]. The success of such schemes will therefore rely on their ability to overcome the psychological, logistical or structural barriers that could limit individuals' engagement within more deprived areas.

A key strength of this paper is that it is based on a nationally representative sample, with a 3-year follow up period. Our analyses controlled for a wide range of potentially confounding factors and were largely consistent across three different measures of area deprivation. However, our study is not without its limitations. First, causality cannot be established given that this is an observational study where other unobserved factors may still confound the association between CCE and mental health/wellbeing. The relatively low levels of model fit and fairly small effect sizes make it worthwhile for further research to test how additional socio-demographic measures not routinely collected by social surveys might shape well-being outcomes. Despite this, a number of previous studies have already confirmed that CCE can be causally linked to wellbeing through randomised interventions [1,2], and the generalisability of such findings has been suggested through causal inference analyses of longitudinal data in other papers [3].

As another limitation, the Index of Multiple Deprivation is comprised of various components (e.g. living environment, income and employment deprivation) as a proxy of neighbourhood effect. But it will be important to understand from future studies how other neighbourhood characteristics (e.g. demographic structure and population density) may also influence the engagement level. Relatedly, future study may be needed to replicate the research on young people's data, given that the moderating effects of area deprivation may be more noticeable during childhood when young people are less able to travel to engage in cultural activities outside of their immediate residential area. Finally, due to data limitations, we were unable to identify whether CCE was recent or more long-standing. It is likely that people who engage in culture and heritage over long periods of time may experience a greater level of mental wellbeing. Future research may also want to explore other mental health measures that are more commonly used in clinical practices, such as the Patient Health Questionnaire (PHQ) and General Anxiety Disorder (GAD) measure.

For peer review only

Conclusion

Our study provides insights into the role of place in long-term relationships between engagement in community cultural assets (both cultural events and museums and heritage sites) and mental wellbeing. Specifically, a higher CCE rate is associated with somewhat greater levels of life satisfaction and mental health functioning and with reduced mental distress. Notably, such relationships are independent of individual demographic and socio-economic characteristics, and CCE is consistently associated with higher wellbeing regardless of where people live. As such, individuals living in areas of high deprivation can experience the same wellbeing benefits from CCE as individuals living in areas of low deprivation. Further, there is also some evidence of moderation, with individuals in areas of high deprivation potentially even able to benefit more from CCE in terms of mental health functioning and improvements in mental distress. However, this does not mitigate the problem that individuals in areas of high deprivation are less likely to engage in CCE. This, therefore, suggests the importance of exploring further the effects of place-based funding schemes that involve investment in areas of higher deprivation to improve engagement rates to confirm if such schemes could help to promote higher levels of wellbeing amongst individuals in such areas. Findings from this study warrant further research in other datasets.

Competing interests

The authors have no conflicts of interest to disclose.

Figure legend

Figure 1 Association between cultural attendance and mental distress by levels of area deprivation. Data: Understanding Society, Waves 2 & 5. Estimates are derived from an OLS regression model including an interaction term (cultural attendance*IMD).

Figure 2 Association between cultural attendance and mental health functioning by levels of area deprivation. Data: Understanding Society, Waves 2 & 5. Estimates are derived from an OLS regression model including an interaction term (cultural attendance*IMD).

Figure 3 Association between museum and heritage engagement and mental health functioning by levels of area deprivation. Data: Understanding Society, Waves 2 & 5. Estimates are derived from an OLS regression model including an interaction term (museum and heritage engagement*IMD).

For peer review only

Funding

DF is supported by the Wellcome Trust [205407/Z/16/Z]. HWM is funded through the AHRC project HEARTS [AH/P005888/1]. This project is also supported by UCL Grand Challenges Award "Environment and Wellbeing" [551987/100/156425 33002] and ESRC WELLCOMM project [ES/T006994/1].

References

1 Grossi E, Tavano GT, Sacco PL. Magic moments: Determinants of stress relief and subjective wellbeing from visiting a cultural heritage site. *Cult Med Psychiatry* 2019;**43**:4–24. doi:10.1007/s11013-018-9593-8

2 Morse N, Thomson LJM, Brown Z, *et al.* Effects of creative museum outreach sessions on measures of confidence, sociability and well-being for mental health and addiction recovery service- users. *Arts Health* 2015;**7**:231–46. doi:10.1080/17533015.2015.1061570

3 Wang S, Mak HW, Fancourt D. Arts, mental distress, mental health functioning & life satisfaction: Fixed-effects analyses of a nationally-representative panel study. *BMC Public Health* 2020;**20**:208. doi:10.1186/s12889-019-8109-y

4 Chatterjee HJ, Noble G. *Museums, Health and Wellbeing*. Farnham, UK; Burlington USA: : Ashgate Publishing Ltd 2013. doi:10.1080/13607863.2013.818101

5 Todd C, Camic PM, Lockyer B, *et al.* Museum-based programs for socially isolated older adults: Understanding what works. *Heal Place* 2017;**48**:47–55. doi:10.1016/j.healthplace.2017.08.005

6 Power A, Smyth K. Heritage, health and place: The legacies of local community-based heritage conservation on social wellbeing. *Heal Place* 2016;**39**:160–7. doi:10.1016/j.healthplace.2016.04.005

7 Bygren LO, Johansson S-E, Konlaan BB, *et al.* Attending cultural events and cancer mortality: A Swedish cohort study. *Arts Health* 2009;**1**:64–73. doi:10.1080/17533010802528058

8 Fancourt D, Steptoe A. The art of life and death: 14 year follow-up analyses of associations between arts engagement and mortality in the English Longitudinal Study of Ageing. *BMJ* 2009;**367**. doi:10.1136/bmj.l6377

9 Roberts S, Camic PM, Springham N. New roles for art galleries: Art-viewing as a community intervention for family carers of people with mental health problems. *Arts Health* 2011;**3**:146–59. doi:10.1080/17533015.2011.561360

10 Shaer D, Beaven K, Springham N, *et al.* The role of art therapy in a pilot for art-based Information Prescriptions at Tate Britain. *Int J Art Ther - Former Inscape* 2008;**13**:25–33. doi:10.1080/17454830802069877

11 Lackoi K, Patsou M, Chatterjee HJ et al. A Preliminary Report from the National Alliance for Museums, Health and Wellbeing. *Museums, Health and Wellbeing* 2016. https://museumsandwellbeingalliance.wordpress.com (accessed 6 Aug 2020).

12 Brook O. Spatial equity and cultural participation: How access influences attendance at museums and galleries in London. *Cult Trends* 2016;**25**:21–34.

For peer review only

13 Mak HW, Coulter R, Fancourt D. Does arts and cultural engagement vary geographically? Evidence from the UK Household Longitudinal Study. *Public Health* 2020;**185**:119–26.

14 Mak HW, Coulter R, Fancourt D. Is area deprivation associated with differential engagement in arts, culture and heritage? Evidence from three nationally-representative samples. n.d.

15 English IMD explorer. 2015. http://dclgapps.communities.gov.uk/imd/idmap.html (accessed 26 Feb 2020).

16 Map of Museums in Britain. https://www.historic-uk.com/HistoryMagazine/DestinationsUK/Museums/ (accessed 1 May 2020).

17 UNESCO World Heritage, United Kingdom of Great Britain and Northern Ireland. UNESCO and World Heritage Convention.https://whc.unesco.org/en/statesparties/gb (accessed 28 Oct 2019).

18 Widdop P, Cutts D. Cultural Trends Impact of place on museum participation. *Cult Trends* 2012;**21**:47–66. doi:10.1080/09548963.2012.641775

19 Houston D, Ong P. Arts accessibility to major museums and cultural/ethnic institutions in Los Angeles: can school tours overcome neighborhood disparities? *Environ Plan A* 2013;**45**:728–48. doi:10.1068/a45206

20 Bourdieu P. *Distinction: a social critique of the judgement of taste*. USA: Cambridge, Massachusetts: Harvard University Press 1984. doi:10.1192/bjp.112.483.211-a

21 Duncan DT, Kawachi I. *Neighborhoods and health*. 2nd ed. New York: : Oxford University Press 2018.

22 Adams RJ, Howard N, Tucker G, *et al.* Effects of area deprivation on health risks and outcomes: a multilevel, cross-sectional, Australian population study. *Int J Public Heal* 2009;**54**:183–92. doi:10.1007/s00038-009-7113-x

23 Macdonald L, Cummins S, Macintyre S. Neighbourhood fast food environment and area deprivation-substitution or concentration? *Appetite* 2007;**49**:251–4. doi:10.1016/j.appet.2006.11.004

24 Stafford M, Marmot M. Neighbourhood deprivation and health: Does it affect us all equally? *Int J Epidemiol* 2003;**32**:357–66. doi:10.1093/ije/dyg084

25 Smith SJ, Easterlow D. The strange geography of health inequalities. *Trans Inst Br Geogr* 2005;**30**:173-90.

26 Smith T, Noble M, Noble S, *et al.* The English Indices of Deprivation 2015 Technical report. 2015. https://assets.publishing.service.gov.uk/government/uploads/system/uploads/attachment_data/file/464485/English_Indices_of_Deprivation_2015_-_Technical-Report.pdf (accessed 14 Aug 2020).

27 Drinkwater C, Wildman J, Moffatt S. Social prescribing. *BMJ* 2019;**364**:l1285. doi:10.1136/bmj.l1285

28 Hilary Bungay SRR A, Clift SB, of Health P. Arts on Prescription: A review of practice in the UK. *Perspect Public Health* 2010;**130**. doi:10.1177/1757913910384050

29 The Social Prescribing Network. *The Social Prescribing Network*. https://www.socialprescribingnetwork.com/ (accessed 28 Oct 2019).

For peer review only

30 Alderwick HAJ, Gottlieb LM, Fichtenberg CM, *et al.* Social prescribing in the U.S. and England: Emerging interventions to address patients' social needs. *Am J Prev Med* 2018;**54**:715–8. doi:10.1016/j.amepre.2018.01.039

31 Jensen A, Stickley T, Torrissen W, *et al.* Arts on prescription in Scandinavia: A review of current practice and future possibilities. *Perspect Public Health* 2017;**137**:268–74. doi:10.1177/1757913916676853

32 Arts Council England. *Creative People and Places*. https://www.artscouncil.org.uk/get-funding/creative-people-and-places (accessed 28 Oct 2019).

33 Buck N, Mcfall S. Understanding Society: Design overview. *Longit Life Course Stud* 2012;**3**:5–17.

34 Office for National Statistics. *Personal well-being in the UK: April 2019 to March 2020*. 2020 .https://www.ons.gov.uk/peoplepopulationandcommunity/wellbeing/bulletins/measuringnationalwellbeing/april2019tomarch2020#measuring-the-data (accessed 27 May 2021).

35 Pevalin DJ. Multiple applications of the GHQ-12 in a general population sample: An investigation of long-term retest effects. *Soc Psychiatry Psychiatr Epidemiol* 2000 Nov;35(11):508-12. doi: 10.1007/s001270050272.

36 Goldberg DP, Williams P. *A user's guide to the General Health Questionnaire*. Windsor: NFER-Nelson 1988. https://www.worldcat.org/title/users-guide-to-the-general-health-questionnaire/oclc/26545847 (accessed 27 May 2021).

37 Ware J, Kosinksi M, Keller S. A 12-Item Short-Form Health Survey: Construction of Scales and Preliminary Tests of Reliability and Validity. *Med Care* 1996;**34**:220–33. doi:10.1097/01.mlr.0000114910.46921.73

38 Kaminska O, Lynn P. Weighting and Sample Representation: Frequently Asked Questions. *Understanding Society: The UK Household Longitudinal Study* 2019. https://www.understandingsociety.ac.uk/sites/default/files/downloads/general/weighting_faqs.pdf (accessed 27 May 2021).

39 Mak HW, Coulter R, Fancourt D. Patterns of social inequality in arts and cultural participation: findings from a nationally-representative sample of adults living in the UK. *WHO Public Heal Panor* 2020;**6**:55–68.

For peer review only

Table 1: Descriptive statistics of the unweighted and weighted sample

	Unweighted (N=22,463)	Weighted (N=14,783)
	Proportion/ Mean (SE)	Proportion/ Mean (SE)
Community cultural engagement, Wave 2		
Cultural events[1]	2.19 (1.47)	2.22 (0.01)
Museums & heritage sites[1]	1.95 (1.50)	1.96 (0.01)
Mental health and wellbeing, Wave 5		
Life satisfaction[2]	5.03 (1.51)	5.06 (0.01)
Mental health distress (GHQ-12, ranging from 1 to 4)	1.93 (0.00)	1.92 (0.00)
Mental health functioning (SF-12; ranging from 1 to 5)	3.74 (0.00)	3.76 (0.01)
Demographic backgrounds, Wave 2		
Age	47.8 (16.9)	46.9 (0.17)
Gender		
Female	56.7%	51.8%
Male	43.3%	48.2%
Ethnicity		
White	86.1%	90.7%
Asian/Asian British	7.89%	5.37%
Black/Black British	3.66%	2.22%
Mixed/other	2.35%	1.76%
Living alone		
No	84.8%	84.8%
Yes	15.2%	15.2%
Partnership status		
Single and never married	17.7%	21.9%
Married or in cohabitation	67.8%	64.3%
Separated or divorced or widowed	14.5%	13.8%
Responsible for child(ren) under 16		
No	80.8%	83.6%
Yes	19.3%	16.4%
Regions		
North (North East, North West and Yorkshire and the Humber)	27.9%	28.2%
Midlands (East Midlands and West Midlands)	19.9%	18.8%
South (London, South East, South West and East)	52.2%	53.1%
Socio-economic position, Wave 2		
Educational levels		

For peer review only

University degree	37.2%	35.2%
Advanced (higher degree/A-level)	19.6%	20.2%
GCSE or equivalent	21.4%	21.3%
Unrecognised/no qualification	21.9%	23.3%
Occupational socio-economic status		
Managerial/professional	28.5%	27.4%
Intermediate/small employment/own account	15.7%	15.5%
lower supervision or lower technician/semi-routine and routine	21.0%	22.5%
Not in employment (incl. retired, full-time student)	34.7%	34.3%
Household monthly gross income		
£0-£1015	21.1%	21.4%
£1015-£1551	24.2%	24.4%
£1551-£2355	26.4%	26.1%
£2355-£32622	28.3%	28.2%
Housing tenure		
House owner	74.2%	72.1%
Social rent	15.4%	15.9%
Private rent	10.5%	12.0%
Levels of area deprivation[3]	5.66 (2.84)	5.70 (0.03)

Notes: [1]A six-point scale, ranging from "not once in the last 12 months", "once in the last 12 months", "twice in the last 12 months", "less often than once a month but at least 3 or 4 times a year", "less often than once a week but at least once a month" to "at least once a week". [2]Life satisfaction was measured using a scale from 1 "completely dissatisfied" to 7 "completely satisfied". [3]Levels of area deprivation was derived from the Index of Multiple Deprivation (IMD), which has a scale from 1 "most deprived 10%" to 10 "least deprived 10%".

For peer review only

Table 2: OLS regression estimating the association between community cultural engagement (CCE) at Wave 2 and life satisfaction at Wave 5 (weighted; N=14,783)

	M1: Basic model			M2: M1 + IMD & interaction terms			M3: M2 + Demographic factors			M4: M3 + Socio-economic position		
	Coef	95%CI	p-value	Coef	95%CI	p-value	Coef	95%CI	p-value	Coef	95%CI	p-value
Cultural events	**0.10**	**0.08 - 0.12**	**0.000**	**0.10**	**0.05 - 0.15**	**0.000**	**0.12**	**0.07 - 0.16**	**0.000**	**0.08**	**0.04 - 0.13**	**0.000**
IMD				**0.07**	**0.05 - 0.09**	**0.000**	**0.06**	**0.04 - 0.08**	**0.000**	**0.04**	**0.02 - 0.06**	**0.000**
Cultural events*IMD				-0.00	-0.01 - 0.00	0.353	-0.00	-0.01 - 0.00	0.300	-0.00	-0.01 - 0.00	0.516
Constant	**4.84**	**4.79 - 4.89**	**0.000**	**4.47**	**4.36 - 4.59**	**0.000**	**4.35**	**4.18 - 4.52**	**0.000**	**4.08**	**3.68 - 4.47**	**0.000**
R-squared	0.01			0.02			0.03			0.04		
Museums & heritage sites	**0.11**	**0.09 - 0.13**	**0.000**	**0.11**	**0.06 - 0.15**	**0.000**	**0.10**	**0.05 - 0.14**	**0.000**	**0.06**	**0.02 - 0.11**	**0.006**
IMD				**0.07**	**0.05 - 0.09**	**0.000**	**0.06**	**0.05 - 0.08**	**0.000**	**0.04**	**0.02 - 0.06**	**0.000**
Museums & heritage sites*IMD				-0.00	-0.01 - 0.00	0.235	-0.00	-0.01 - 0.00	0.267	-0.00	-0.01 - 0.00	0.618
Constant	**4.85**	**4.80 - 4.90**	**0.000**	**4.50**	**4.40 - 4.61**	**0.000**	**4.49**	**4.33 - 4.65**	**0.000**	**4.16**	**3.77 - 4.54**	**0.000**
R-squared	0.01			0.02			0.03			0.04		

Note: M1 (basic model) included CCE. M2 additionally controlled for Index of Multiple Deprivation (IMD) and interaction terms (CCE*IMD). M3 additionally adjusted for demographic factors (age, gender, ethnicity, marital status, presence of children under age 16, whether or not living alone, regional locations). M4 additionally adjusted for socio-economic position (educational levels, occupational socio-economic status, household monthly gross income, and housing tenure). Bold values denote statistical significance at the p < 0.05 level.

For peer review only

Table 3: OLS regression estimating the association between community cultural engagement (CCE) at Wave 2 and mental distress (GHQ-12) at Wave 5 (weighted; N=14,783)

	M1: Basic model			M2: M1 + IMD & interaction terms			M3: M2 + Demographic factors			M4: M3 + Socio-economic position		
	Coef	95%CI	p-value	Coef	95%CI	p-value	Coef	95%CI	p-value	Coef	95%CI	p-value
Cultural events	**-0.02**	**-0.03 - -0.02**	**0.000**	**-0.03**	**-0.05 - -0.02**	**0.000**	**-0.04**	**-0.05 - -0.02**	**0.000**	**-0.03**	**-0.04 - -0.01**	**0.000**
IMD				**-0.02**	**-0.03 - -0.01**	**0.000**	**-0.02**	**-0.02 - -0.01**	**0.000**	**-0.01**	**-0.02 - -0.01**	**0.000**
Cultural events*IMD				**0.00**	**0.00 - 0.00**	**0.012**	**0.00**	**0.00 - 0.00**	**0.033**	0.00	-0.00 - 0.00	0.107
Constant	**1.97**	**1.96 - 1.99**	**0.000**	**2.07**	**2.04 - 2.11**	**0.000**	**2.11**	**2.05 - 2.16**	**0.000**	**2.21**	**2.09 - 2.32**	**0.000**
R-squared	0.01			0.01			0.03			0.04		
Museums & heritage sites	**-0.02**	**-0.03 - -0.02**	**0.000**	**-0.03**	**-0.04 - -0.01**	**0.000**	**-0.03**	**-0.04 - -0.01**	**0.000**	**-0.02**	**-0.03 - -0.00**	**0.020**
IMD				**-0.02**	**-0.02 - -0.01**	**0.000**	**-0.02**	**-0.02 - -0.01**	**0.000**	**-0.01**	**-0.02 - -0.01**	**0.000**
Museums & heritage sites*IMD				0.00	-0.00 - 0.00	0.106	0.00	-0.00 - 0.00	0.101	0.00	-0.00 - 0.00	0.425

For peer review only

Constant	**1.97**	**1.95 - 1.98**	**0.000**	**2.05**	**2.02 - 2.08**	**0.000**	**2.06**	**2.01 - 2.11**	**0.000**	**2.17**	**2.06 - 2.29**	**0.000**
R-squared	0.01			0.01			0.03			0.04		

Note: M1 (basic model) included CCE. M2 additionally controlled for Index of Multiple Deprivation (IMD) and interaction terms (CCE*IMD). M3 additionally adjusted for demographic factors (age, gender, ethnicity, marital status, presence of children under age 16, whether or not living alone, regional locations). M4 additionally adjusted for socio-economic position (educational levels, occupational socio-economic status, household monthly gross income, and housing tenure). Bold values denote statistical significance at the $p < 0.05$ level.

For peer review only

Table 4: OLS regression estimating the association between community cultural engagement (CCE) at Wave 2 and mental health functioning (SF-12) at Wave 5 (weighted; N=14,783)

	M1: Basic model	M2: M1 + IMD & interaction	M3: M2 + Demographic factors	M4: M3 + Socio-economic

					terms						position	
	Coef	95%CI	p-value	Coef	95%CI	p-value	Coef	95%CI	p-value	Coef	95%CI	p-value
Cultural events	**0.10**	**0.10 - 0.11**	**0.000**	**0.12**	**0.10 - 0.14**	**0.000**	**0.10**	**0.08 - 0.12**	**0.000**	**0.06**	**0.04 - 0.08**	**0.000**
IMD				**0.05**	**0.04 - 0.05**	**0.000**	**0.05**	**0.04 - 0.05**	**0.000**	**0.03**	**0.02 - 0.04**	**0.000**
Cultural events*IMD				**-0.01**	**-0.01 - -0.00**	**0.000**	**-0.00**	**-0.01 - -0.00**	**0.008**	-0.00	-0.00 - 0.00	0.183
Constant	**3.53**	**3.50 - 3.55**	**0.000**	**3.30**	**3.25 - 3.35**	**0.000**	**3.76**	**3.68 - 3.83**	**0.000**	**3.68**	**3.51 - 3.84**	**0.000**
R-squared	0.05			0.07			0.12			0.16		
Museums & heritage sites	**0.08**	**0.07 - 0.09**	**0.000**	**0.09**	**0.07 - 0.11**	**0.000**	**0.08**	**0.06 - 0.10**	**0.000**	**0.05**	**0.03 - 0.06**	**0.000**
IMD				**0.04**	**0.03 - 0.05**	**0.000**	**0.04**	**0.03 - 0.05**	**0.000**	**0.02**	**0.02 - 0.03**	**0.000**
Museums & heritage sites*IMD				**-0.00**	**-0.01 - -0.00**	**0.027**	-0.00	-0.01 - 0.00	0.086	0.00	-0.00 - 0.00	0.948
Constant	**3.60**	**3.57 - 3.62**	**0.000**	**3.40**	**3.35 - 3.45**	**0.000**	**3.87**	**3.80 - 3.94**	**0.000**	**3.74**	**3.58 - 3.90**	**0.000**
R-squared	0.03			0.05			0.12			0.16		

Note: M1 (basic model) included CCE. M2 additionally controlled for Index of Multiple Deprivation (IMD) and interaction terms (CCE*IMD). M3 additionally adjusted for demographic factors (age, gender, ethnicity, marital status, presence of children under age 16, whether or not living alone, regional locations). M4 additionally adjusted for socio-economic position (educational levels, occupational socio-economic status, household monthly gross income, and housing tenure). Bold values denote statistical significance at the p < 0.05 level.

For peer review only

For peer review only

For peer review only

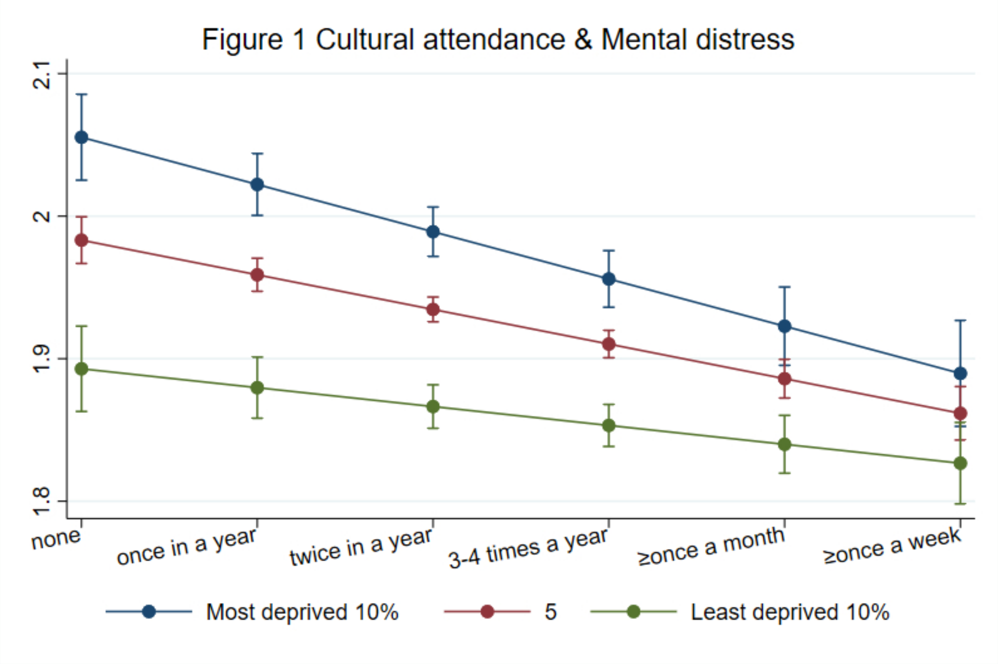

Figure 1 Cultural attendance & Mental distress

281x187mm (72 x 72 DPI)

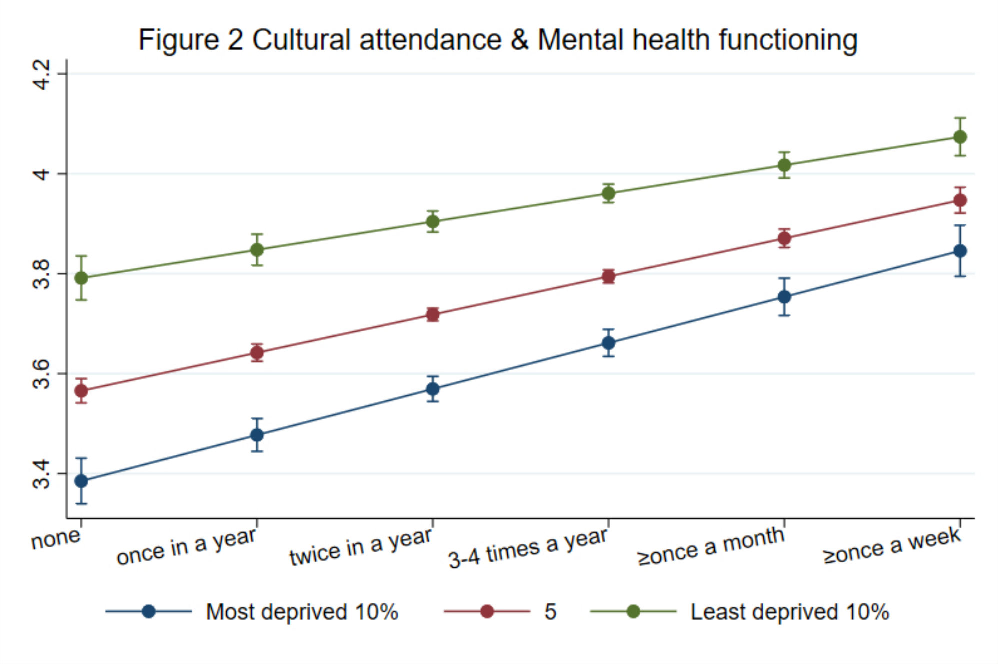

Figure 2 Cultural attendance & Mental health functioning

281x187mm (72 x 72 DPI)

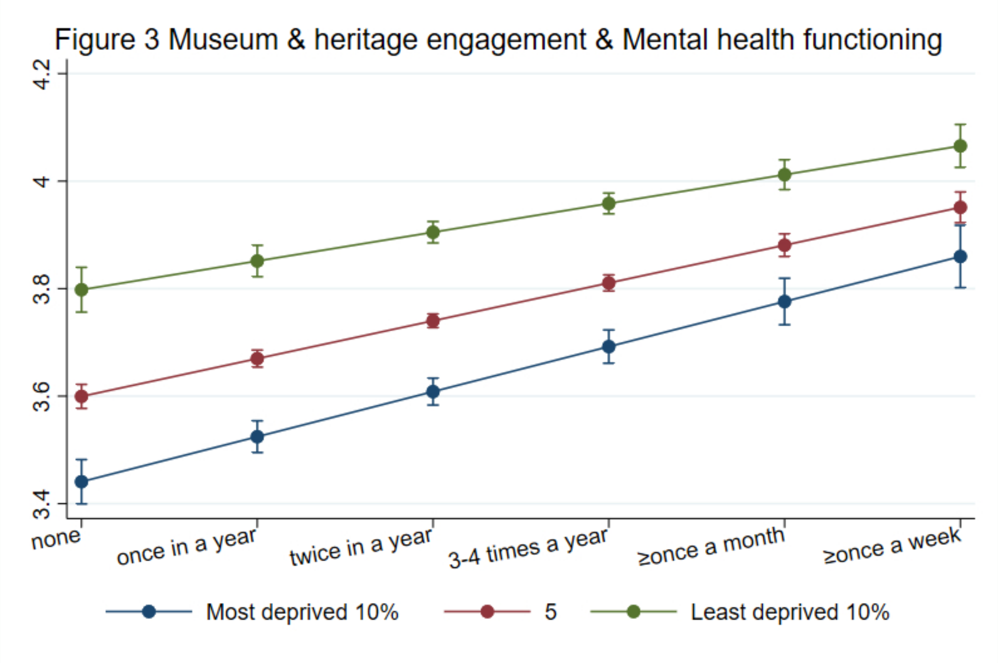

Figure 3 Museum & heritage engagement & Mental health functioning

281x187mm (72 x 72 DPI)

Appendix I

Cultural events

- A film at a cinema or other venue
- Exhibition or collection or art, photography or sculpture or a craft exhibition (not craft market)
- Event which included video or electronic art
- Event connected with books or writing
- Street arts or a public art display or installation (art in everyday surroundings, or an art work such as sculpture that is outdoors or in a public place)
- Carnival or culturally specific festival (for example, Mela, Baisakhi, Navrati, Feis)
- Circus (not animals)
- Play/drama, pantomime or a musical
- Opera/operetta
- Classical music performance
- Rock, pop or jazz performance
- Ballet
- Contemporary dance
- African people's dance or south Asian and Chinese dance

Museums/galleries and heritage sites visits

- Visit a museum or gallery
- Visit any of the following heritage sites:
  - A city or town with historic character
  - A historic building open to the public (non-religious)
  - A historic park or garden open to the public
  - A place connected with industrial history (e.g. an old factory, dockyard or mine) or historic transport system (e.g. an old ship or railway)
  - A historic place of worship attended as a visitor (not to worship)
  - A monument such as a castle, fort or ruin
  - A site of archaeological interest (e.g. Roman villa, ancient burial site)
  - A site connected with sports heritage (e.g. Wimbledon) (not visited for the purposes of watching sport)

For peer review only

Supplementary

STable 1: OLS regression estimating the association between community cultural engagement (CCE) at Wave 2 and life satisfaction at Wave 5 (weighted; N=14,783)

	M1: Basic model			M2: M1 + IMD & interaction terms			M3: M2 + Demographic factors			M4: M3 + Socio-economic position		
	Coef	95%CI	p-value	Coef	95%CI	p-value	Coef	95%CI	p-value	Coef	95%CI	p-value
Cultural events	**0.10**	**0.08 - 0.12**	**0.000**	**0.10**	**0.07 - 0.12**	**0.000**	**0.11**	**0.09 - 0.14**	**0.000**	**0.08**	**0.06 - 0.11**	**0.000**
20% most deprived area				**-0.32**	**-0.45 - -0.18**	**0.000**	**-0.26**	**-0.40 - -0.13**	**0.000**	**-0.16**	**-0.29 - -0.02**	**0.023**
20% least deprived area				**0.24**	**0.10 - 0.38**	**0.001**	**0.21**	**0.08 - 0.35**	**0.002**	**0.15**	**0.01 - 0.29**	**0.033**
(ref: medium levels of deprivation)												
Cultural events*20% most deprived area				-0.03	-0.08 - 0.03	0.353	-0.02	-0.08 - 0.03	0.443	-0.02	-0.07 - 0.04	0.485
Cultural events*20% least deprived area				-0.03	-0.08 - 0.02	0.264	-0.03	-0.08 - 0.02	0.203	-0.02	-0.07 - 0.03	0.352
Constant	**4.84**	**4.79 - 4.89**	**0.000**	**4.88**	**4.82 - 4.95**	**0.000**	**4.68**	**4.54 - 4.82**	**0.000**	**4.28**	**3.90 - 4.66**	**0.000**
R-squared	0.01			0.02			0.03			0.04		
Museums & heritage sites	**0.11**	**0.09 - 0.13**	**0.000**	**0.09**	**0.07 - 0.11**	**0.000**	**0.08**	**0.06 - 0.11**	**0.000**	**0.06**	**0.03 - 0.08**	**0.000**
20% most deprived area				**-0.34**	**-0.46 - -0.22**	**0.000**	**-0.30**	**-0.43 - -0.18**	**0.000**	**-0.19**	**-0.31 - -0.06**	**0.003**
20% least deprived area				**0.20**	**0.07 - 0.33**	**0.002**	**0.18**	**0.05 - 0.31**	**0.005**	0.11	-0.01 - 0.24	0.081
(ref: medium levels of deprivation)												
Museums & heritage sites*20% most deprived area				-0.01	-0.06 - 0.05	0.782	-0.01	-0.06 - 0.05	0.843	-0.00	-0.06 - 0.05	0.862
Museums & heritage sites*20% least deprived area				-0.02	-0.06 - 0.03	0.518	-0.02	-0.06 - 0.03	0.517	-0.01	-0.05 - 0.04	0.799
Constant	**4.85**	**4.80 - 4.90**	**0.000**	**4.91**	**4.85 - 4.97**	**0.000**	**4.85**	**4.72 - 4.98**	**0.000**	**4.36**	**3.98 - 4.74**	**0.000**
R-squared	0.01			0.02			0.03			0.04		

Note: M1 (basic model) included CCE. M2 additionally controlled for Index of Multiple Deprivation (IMD) and interaction terms (CCE*IMD). M3 additionally adjusted for demographic factors (age, gender, ethnicity, marital status, presence of children under age 16, whether or not living alone, regional locations). M4 additionally adjusted for socio-economic position (educational levels, occupational socio-economic status, household monthly gross income, and housing tenure). Bold values denote statistical significance at the p < 0.05 level.

For peer review only

STable 2: OLS regression estimating the association between community cultural engagement (CCE) at Wave 2 and life satisfaction at Wave 5 (weighted; N=14,783)

	M1: Basic model			M2: M1 + IMD & interaction terms			M3: M2 + Demographic factors			M4: M3 + Socio-economic position		
	Coef	95%CI	p-value	Coef	95%CI	p-value	Coef	95%CI	p-value	Coef	95%CI	p-value
Cultural events	0.10	0.08 - 0.12	0.000	**0.10**	**0.08 - 0.12**	**0.000**	**0.11**	**0.09 - 0.13**	**0.000**	**0.08**	**0.06 - 0.10**	**0.000**
10% most deprived area				**-0.35**	**-0.53 - -0.17**	**0.000**	**-0.28**	**-0.46 - -0.10**	**0.002**	-0.16	-0.34 - 0.02	0.076
10% least deprived area				**0.28**	**0.09 - 0.47**	**0.004**	**0.26**	**0.07 - 0.45**	**0.008**	0.17	-0.02 - 0.37	0.074
(ref: medium levels of deprivation)												
Cultural events*10% most deprived area				-0.05	-0.14 - 0.03	0.217	-0.05	-0.13 - 0.03	0.251	-0.04	-0.12 - 0.04	0.348
Cultural events*10% least deprived area				-0.03	-0.09 - 0.04	0.418	-0.03	-0.10 - 0.03	0.281	-0.02	-0.09 - 0.04	0.471
Constant	4.84	4.79 - 4.89	0.000	**4.86**	**4.81 - 4.92**	**0.000**	**4.66**	**4.52 - 4.79**	**0.000**	**4.25**	**3.87 - 4.63**	**0.000**
R-squared	0.01			0.02			0.03			0.04		
Museums & heritage sites	0.11	0.09 - 0.13	0.000	**0.09**	**0.07 - 0.11**	**0.000**	**0.08**	**0.06 - 0.10**	**0.000**	**0.05**	**0.03 - 0.07**	**0.000**
10% most deprived area				**-0.41**	**-0.57 - -0.25**	**0.000**	**-0.38**	**-0.54 - -0.21**	**0.000**	**-0.24**	**-0.40 - -0.07**	**0.004**
10% least deprived area				**0.18**	**0.00 - 0.37**	**0.048**	0.16	-0.02 - 0.34	0.081	0.07	-0.11 - 0.25	0.462
(ref: medium levels of deprivation)												
Museums & heritage sites*10% most deprived area				-0.00	-0.08 - 0.07	0.921	-0.00	-0.08 - 0.08	0.981	0.00	-0.07 - 0.08	0.902
Museums & heritage sites*10% least deprived area				0.01	-0.05 - 0.07	0.789	0.01	-0.05 - 0.07	0.807	0.02	-0.04 - 0.08	0.505
Constant	4.85	4.80 - 4.90	0.000	**4.90**	**4.84 - 4.95**	**0.000**	**4.83**	**4.71 - 4.96**	**0.000**	**4.34**	**3.96 - 4.72**	**0.000**
R-squared	0.01			0.02			0.03			0.04		

Note: M1 (basic model) included CCE. M2 additionally controlled for Index of Multiple Deprivation (IMD) and interaction terms (CCE*IMD). M3 additionally adjusted for demographic factors (age, gender, ethnicity, marital status, presence of children under age 16, whether or not living alone, regional locations). M4 additionally adjusted for socio-economic position (educational levels, occupational socio-economic status, household monthly gross income, and housing tenure). Bold values denote statistical significance at the p < 0.05 level.

For peer review only

STable 3: OLS regression estimating the association between community cultural engagement (CCE) at Wave 2 and mental distress (GHQ-12) at Wave 5 (weighted; N=14,783)

	M1: Basic model			M2: M1 + IMD & interaction terms			M3: M2 + Demographic factors			M4: M3 + Socio-economic position		
	Coef	95%CI	p-value	Coef	95%CI	p-value	Coef	95%CI	p-value	Coef	95%CI	p-value
Cultural events	**-0.02**	**-0.03 - -0.02**	**0.000**	**-0.02**	**-0.02 - -0.01**	**0.000**	**-0.02**	**-0.03 - -0.02**	**0.000**	**-0.02**	**-0.02 - -0.01**	**0.000**
20% most deprived area				**0.09**	**0.05 - 0.13**	**0.000**	**0.08**	**0.04 - 0.12**	**0.000**	**0.05**	**0.01 - 0.09**	**0.012**
20% least deprived area				**-0.07**	**-0.11 - -0.03**	**0.000**	**-0.07**	**-0.11 - -0.03**	**0.001**	**-0.05**	**-0.09 - -0.01**	**0.007**
(ref: medium levels of deprivation)												
Cultural events*20% most deprived area				-0.01	-0.03 - 0.00	0.070	-0.01	-0.03 - 0.00	0.105	-0.01	-0.03 - 0.00	0.118
Cultural events*20% least deprived area				0.01	-0.01 - 0.02	0.383	0.01	-0.01 - 0.02	0.376	0.00	-0.01 - 0.02	0.613
Constant	**1.97**	**1.96 - 1.99**	**0.000**	**1.96**	**1.94 - 1.98**	**0.000**	**2.01**	**1.96 - 2.05**	**0.000**	**2.14**	**2.03 - 2.25**	**0.000**
R-squared	0.01			0.01			0.03			0.04		
Museums & heritage sites	**-0.02**	**-0.03 - -0.02**	**0.000**	**-0.02**	**-0.02 - -0.01**	**0.000**	**-0.01**	**-0.02 - -0.01**	**0.000**	**-0.01**	**-0.02 - -0.00**	**0.013**
20% most deprived area				**0.08**	**0.04 - 0.12**	**0.000**	**0.08**	**0.04 - 0.12**	**0.000**	**0.05**	**0.01 - 0.08**	**0.014**
20% least deprived area				**-0.06**	**-0.10 - -0.02**	**0.002**	**-0.06**	**-0.10 - -0.02**	**0.002**	**-0.04**	**-0.08 - -0.01**	**0.025**
(ref: medium levels of deprivation)												
Museums & heritage sites*20% most deprived area				-0.01	-0.03 - 0.00	0.108	-0.01	-0.03 - 0.00	0.096	-0.01	-0.03 - 0.00	0.151
Museums & heritage sites*20% least deprived area				0.00	-0.01 - 0.01	0.924	0.00	-0.01 - 0.01	0.862	-0.00	-0.02 - 0.01	0.758
Constant	**1.97**	**1.95 - 1.98**	**0.000**	**1.95**	**1.93 - 1.97**	**0.000**	**1.97**	**1.93 - 2.00**	**0.000**	**2.12**	**2.01 - 2.23**	**0.000**
R-squared	0.01			0.01			0.03			0.04		

Note: M1 (basic model) included CCE. M2 additionally controlled for Index of Multiple Deprivation (IMD) and interaction terms (CCE*IMD). M3 additionally adjusted for demographic factors (age, gender, ethnicity, marital status, presence of children under age 16, whether or not living alone, regional locations). M4 additionally adjusted for socio-economic position (educational levels, occupational socio-economic status, household monthly gross income, and housing tenure). Bold values denote statistical significance at the $p < 0.05$ level.

For peer review only

STable 4: OLS regression estimating the association between community cultural engagement (CCE) at Wave 2 and mental distress (GHQ-12) at Wave 5 (weighted; N=14,783)

	M1: Basic model			M2: M1 + IMD & interaction terms			M3: M2 + Demographic factors			M4: M3 + Socio-economic position		
	Coef	95%CI	p-value	Coef	95%CI	p-value	Coef	95%CI	p-value	Coef	95%CI	p-value
Cultural events	**-0.02**	**-0.03 - -0.02**	**0.000**	**-0.02**	**-0.03 - -0.01**	**0.000**	**-0.03**	**-0.03 - -0.02**	**0.000**	**-0.02**	**-0.03 - -0.01**	**0.000**
10% most deprived area				**0.09**	**0.04 - 0.15**	**0.001**	**0.08**	**0.02 - 0.13**	**0.005**	0.04	-0.01 - 0.10	0.118
10% least deprived area				**-0.10**	**-0.15 - -0.04**	**0.001**	**-0.09**	**-0.15 - -0.04**	**0.001**	**-0.07**	**-0.12 - -0.01**	**0.013**
(ref: medium levels of deprivation)												
Cultural events*10% most deprived area				0.00	-0.02 - 0.02	0.883	0.00	-0.02 - 0.03	0.702	0.00	-0.02 - 0.03	0.714
Cultural events*10% least deprived area				0.01	-0.01 - 0.03	0.155	0.01	-0.00 - 0.03	0.150	0.01	-0.01 - 0.03	0.326
Constant	**1.97**	**1.96 - 1.99**	**0.000**	**1.97**	**1.95 - 1.98**	**0.000**	**2.02**	**1.98 - 2.06**	**0.000**	**2.15**	**2.04 - 2.26**	**0.000**
R-squared	0.01			0.01			0.03			0.04		
Museums & heritage sites	**-0.02**	**-0.03 - -0.02**	**0.000**	**-0.02**	**-0.02 - -0.01**	**0.000**	**-0.02**	**-0.02 - -0.01**	**0.000**	**-0.01**	**-0.02 - -0.00**	**0.001**
10% most deprived area				**0.12**	**0.07 - 0.17**	**0.000**	**0.11**	**0.06 - 0.16**	**0.000**	**0.07**	**0.02 - 0.12**	**0.004**
10% least deprived area				**-0.08**	**-0.13 - -0.02**	**0.006**	**-0.08**	**-0.13 - -0.02**	**0.004**	**-0.05**	**-0.11 - -0.00**	**0.044**
(ref: medium levels of deprivation)												
Museums & heritage sites*10% most deprived area				-0.02	-0.04 - 0.01	0.137	-0.02	-0.04 - 0.00	0.113	-0.02	-0.04 - 0.01	0.140
Museums & heritage sites*10% least deprived area				0.01	-0.01 - 0.02	0.521	0.01	-0.01 - 0.03	0.430	0.00	-0.01 - 0.02	0.734
Constant	**1.97**	**1.95 - 1.98**	**0.000**	**1.95**	**1.94 - 1.97**	**0.000**	**1.97**	**1.93 - 2.01**	**0.000**	**2.12**	**2.01 - 2.23**	**0.000**
R-squared	0.01			0.01			0.02			0.04		

Note: M1 (basic model) included CCE. M2 additionally controlled for Index of Multiple Deprivation (IMD) and interaction terms (CCE*IMD). M3 additionally adjusted for demographic factors (age, gender, ethnicity, marital status, presence of children under age 16, whether or not living alone, regional locations). M4 additionally adjusted for socio-economic position (educational levels, occupational socio-economic status, household monthly gross income, and housing tenure). Bold values denote statistical significance at the p < 0.05 level.

For peer review only

STable 5: OLS regression estimating the association between community cultural engagement (CCE) at Wave 2 and mental health functioning (SF-12) at Wave 5 (weighted; N=14,783)

	M1: Basic model			M2: M1 + IMD & interaction terms			M3: M2 + Demographic factors			M4: M3 + Socio-economic position		
	Coef	95%CI	p-value	Coef	95%CI	p-value	Coef	95%CI	p-value	Coef	95%CI	p-value
Cultural events	**0.10**	**0.10 - 0.11**	**0.000**	**0.10**	**0.09 - 0.11**	**0.000**	**0.08**	**0.07 - 0.09**	**0.000**	**0.05**	**0.04 - 0.06**	**0.000**
20% most deprived area				**-0.18**	**-0.24 - -0.12**	**0.000**	**-0.19**	**-0.25 - -0.13**	**0.000**	**-0.10**	**-0.16 - -0.04**	**0.001**
20% least deprived area				**0.17**	**0.11 - 0.24**	**0.000**	**0.16**	**0.10 - 0.22**	**0.000**	**0.10**	**0.04 - 0.16**	**0.001**
(ref: medium levels of deprivation)												
Cultural events*20% most deprived area				0.01	-0.01 - 0.03	0.380	0.01	-0.01 - 0.03	0.463	0.01	-0.01 - 0.03	0.505
Cultural events*20% least deprived area				**-0.02**	**-0.04 - -0.00**	**0.033**	-0.01	-0.03 - 0.01	0.217	-0.00	-0.02 - 0.02	0.890
Constant	**3.53**	**3.50 - 3.55**	**0.000**	**3.55**	**3.51 - 3.58**	**0.000**	**4.01**	**3.95 - 4.06**	**0.000**	**3.81**	**3.66 - 3.97**	**0.000**
R-squared	0.05			0.07			0.12			0.16		
Museums & heritage sites	**0.08**	**0.07 - 0.09**	**0.000**	**0.07**	**0.06 - 0.09**	**0.000**	**0.07**	**0.06 - 0.08**	**0.000**	**0.05**	**0.04 - 0.06**	**0.000**
20% most deprived area				**-0.16**	**-0.21 - -0.10**	**0.000**	**-0.17**	**-0.22 - -0.11**	**0.000**	**-0.07**	**-0.13 - -0.02**	**0.008**
20% least deprived area				**0.16**	**0.10 - 0.22**	**0.000**	**0.16**	**0.10 - 0.21**	**0.000**	**0.10**	**0.05 - 0.16**	**0.000**
(ref: medium levels of deprivation)												
Museums & heritage sites*20% most deprived area				-0.00	-0.02 - 0.02	0.989	-0.00	-0.03 - 0.02	0.760	-0.01	-0.03 - 0.01	0.419
Museums & heritage sites*20% least deprived area				-0.02	-0.04 - 0.00	0.072	-0.01	-0.03 - 0.01	0.190	-0.00	-0.02 - 0.02	0.805
Constant	**3.60**	**3.57 - 3.62**	**0.000**	**3.62**	**3.59 - 3.64**	**0.000**	**4.10**	**4.04 - 4.15**	**0.000**	**3.84**	**3.68 - 4.00**	**0.000**
R-squared	0.03			0.05			0.11			0.16		

Note: M1 (basic model) included CCE. M2 additionally controlled for Index of Multiple Deprivation (IMD) and interaction terms (CCE*IMD). M3 additionally adjusted for demographic factors (age, gender, ethnicity, marital status, presence of children under age 16, whether or not living alone, regional locations). M4 additionally adjusted for socio-economic position (educational levels, occupational socio-economic status, household monthly gross income, and housing tenure). Bold values denote statistical significance at the p < 0.05 level.

For peer review only

STable 6: OLS regression estimating the association between community cultural engagement (CCE) at Wave 2 and mental health functioning (SF-12) at Wave 5 (weighted; N=14,783)

	M1: Basic model			M2: M1 + IMD & interaction terms			M3: M2 + Demographic factors			M4: M3 + Socio-economic position		
	Coef	95%CI	p-value	Coef	95%CI	p-value	Coef	95%CI	p-value	Coef	95%CI	p-value
Cultural events	**0.10**	**0.10 - 0.11**	**0.000**	**0.10**	**0.09 - 0.11**	**0.000**	**0.08**	**0.07 - 0.09**	**0.000**	**0.05**	**0.04 - 0.06**	**0.000**
10% most deprived area				**-0.21**	**-0.29 - -0.13**	**0.000**	**-0.21**	**-0.29 - -0.13**	**0.000**	**-0.11**	**-0.19 - -0.03**	**0.006**
10% least deprived area				**0.24**	**0.16 - 0.32**	**0.000**	**0.22**	**0.14 - 0.29**	**0.000**	**0.14**	**0.06 - 0.21**	**0.000**
(ref: medium levels of deprivation)												
Cultural events*10% most deprived area				-0.01	-0.04 - 0.03	0.693	-0.01	-0.04 - 0.02	0.500	-0.01	-0.04 - 0.02	0.597
Cultural events*10% least deprived area				**-0.04**	**-0.06 - -0.01**	**0.004**	**-0.03**	**-0.05 - -0.00**	**0.043**	-0.01	-0.03 - 0.02	0.484
Constant	**3.53**	**3.50 - 3.55**	**0.000**	**3.54**	**3.51 - 3.57**	**0.000**	**3.99**	**3.93 - 4.04**	**0.000**	**3.80**	**3.64 - 3.96**	**0.000**
R-squared	0.05			0.07			0.11			0.16		
Museums & heritage sites	**0.08**	**0.07 - 0.09**	**0.000**	**0.07**	**0.07 - 0.08**	**0.000**	**0.07**	**0.06 - 0.08**	**0.000**	**0.05**	**0.04 - 0.06**	**0.000**
10% most deprived area				**-0.23**	**-0.30 - -0.16**	**0.000**	**-0.24**	**-0.31 - -0.17**	**0.000**	**-0.12**	**-0.19 - -0.05**	**0.001**
10% least deprived area				**0.21**	**0.13 - 0.28**	**0.000**	**0.20**	**0.13 - 0.27**	**0.000**	**0.12**	**0.06 - 0.18**	**0.000**
(ref: medium levels of deprivation)												
Museums & heritage sites*10% most deprived area				0.00	-0.03 - 0.04	0.899	0.00	-0.03 - 0.04	0.818	0.00	-0.03 - 0.03	0.958
Museums & heritage sites*10% least deprived area				**-0.03**	**-0.05 - -0.00**	**0.034**	-0.02	-0.04 - 0.00	0.111	-0.00	-0.03 - 0.02	0.769
Constant	**3.60**	**3.57 - 3.62**	**0.000**	**3.61**	**3.59 - 3.64**	**0.000**	**4.09**	**4.04 - 4.14**	**0.000**	**3.83**	**3.68 - 3.99**	**0.000**
R-squared	0.03			0.05			0.11			0.16		

Note: M1 (basic model) included CCE. M2 additionally controlled for Index of Multiple Deprivation (IMD) and interaction terms (CCE*IMD). M3 additionally adjusted for demographic factors (age, gender, ethnicity, marital status, presence of children under age 16, whether or not living alone, regional locations). M4 additionally adjusted for socio-economic position (educational levels, occupational socio-economic status, household monthly gross income, and housing tenure). Bold values denote statistical significance at the p < 0.05 level.

For peer review only

STROBE Statement—Checklist of items that should be included in reports of *cross-sectional studies*		
	**Recommendation**	**Page/Line number**
**Title and abstract**		
	(*a*) Indicate the study's design with a commonly used term in the title or the abstract	P1
	(*b*) Provide in the abstract an informative and balanced summary of what was done and what was found	P2
**Introduction**		
Background/rationale	Explain the scientific background and rationale for the investigation being reported	P4
Objectives	State specific objectives, including any prespecified hypotheses	P5
**Methods**		
Study design	Present key elements of study design early in the paper	P5
Setting	Describe the setting, locations, and relevant dates, including periods of recruitment, exposure, follow-up, and data collection	P5
Participants	(*a*) Give the eligibility criteria, and the sources and methods of selection of participants	P5
Variables	Clearly define all outcomes, exposures, predictors, potential confounders, and effect modifiers. Give diagnostic criteria, if applicable	P5-6
Data sources/ measurement	* For each variable of interest, give sources of data and details of methods of assessment (measurement). Describe comparability of assessment methods if there is more than one group	P5-6
Bias	Describe any efforts to address potential sources of bias	P7
Study size	Explain how the study size was arrived at	P7
Quantitative variables	Explain how quantitative variables were handled in the analyses. If applicable, describe which groupings were chosen and why	P5-6
Statistical methods	(*a*) Describe all statistical methods, including those used to control for confounding	P6-7
	(*b*) Describe any methods used to examine subgroups and interactions	P6-7
	(*c*) Explain how missing data were addressed	P7
	(*d*) If applicable, describe analytical methods taking account of sampling strategy	NA
	(e) Describe any sensitivity analyses	P7
**Results**		

For peer review only

Participants	(a) Report numbers of individuals at each stage of study—eg numbers potentially eligible, examined for eligibility, confirmed eligible, included in the study, completing follow-up, and analysed	P7
	(b) Give reasons for non-participation at each stage	P7
	(c) Consider use of a flow diagram	NA
Descriptive data	(a) Give characteristics of study participants (eg demographic, clinical, social) and information on exposures and potential confounders	P7
	(b) Indicate number of participants with missing data for each variable of interest	P7
Outcome data	Report numbers of outcome events or summary measures	P7 & Table 1
Main results	(*a*) Give unadjusted estimates and, if applicable, confounder-adjusted estimates and their precision (eg, 95% confidence interval). Make clear which confounders were adjusted for and why they were included	P7-8, Tables 2-4
	(*b*) Report category boundaries when continuous variables were categorized	NA
	(*c*) If relevant, consider translating estimates of relative risk into absolute risk for a meaningful time period	NA
Other analyses	Report other analyses done—eg analyses of subgroups and interactions, and sensitivity analyses	P8
**Discussion**		
Key results	Summarise key results with reference to study objectives	P8-9
Limitations	Discuss limitations of the study, taking into account sources of potential bias or imprecision. Discuss both direction and magnitude of any potential bias	P10
Interpretation	Give a cautious overall interpretation of results considering objectives, limitations, multiplicity of analyses, results from similar studies, and other relevant evidence	P8-10
Generalisability	Discuss the generalisability (external validity) of the study results	P10 where we stated that our analysis was based on a nationally representative sample
**Other information**		

For peer review only

Funding	Give the source of funding and the role of the funders for the present study and, if applicable, for the original study on which the present article is based	P3 – Acknowledgement

For peer review only
